# A systematic exploration of the interactions between bacterial effector proteins and host cell membranes

**DOI:** 10.1038/s41467-017-00700-7

**Published:** 2017-09-14

**Authors:** Bethany A. Weigele, Robert C. Orchard, Alyssa Jimenez, Gregory W. Cox, Neal M. Alto

**Affiliations:** 1 Department of Microbiology, University of Texas Southwestern Medical Center, 5323 Harry Hines Boulevard, Dallas, TX 75390-8816 USA; 2 Department of Pathology and Immunology, Washington University School of Medicine, 660 South Euclid Avenue, Saint Louis, MO 63110 USA

## Abstract

Membrane-bound organelles serve as platforms for the assembly of multi-protein complexes that function as hubs of signal transduction in eukaryotic cells. Microbial pathogens have evolved virulence factors that reprogram these host signaling responses, but the underlying molecular mechanisms are poorly understood. Here we test the ability of ~200 type III and type IV effector proteins from six Gram-negative bacterial species to interact with the eukaryotic plasma membrane and intracellular organelles. We show that over 30% of the effectors localize to yeast and mammalian cell membranes, including a subset of previously uncharacterized *Legionella* effectors that appear to be able to regulate yeast vacuolar fusion. A combined genetic, cellular, and biochemical approach supports that some of the tested bacterial effectors can bind to membrane phospholipids and may regulate membrane trafficking. Finally, we show that the type III effector IpgB1 from *Shigella flexneri* may bind to acidic phospholipids and regulate actin filament dynamics.

## Abstract (web summary)

Microbial pathogens secrete effector proteins into host cells to affect cellular functions. Here, the authors use a yeast-based screen to study around 200 effectors from six bacterial species, showing that over 30% of them interact with the eukaryotic plasma membrane or intracellular organelles.

## Introduction

Protein injection systems of Gram-negative bacterial pathogens are among the most thoroughly studied microbial virulence determinants. Although each of the three systems are evolutionarily related to intrinsic molecular machines of microbes including flagellum (i.e., type III), the conjugation pili (i.e., type IV), and phage tail spike apparatus (i.e., type VI), they all function to deliver bacterial effector proteins directly into the host cells^1–3^. Once inside the animal or plant cell, these effector proteins post-translationally modify or allosterically regulate molecules involved in signal transduction or cellular architecture^4^. Despite significant advances in effector protein biochemistry over the past decade^5–7^, much less is known about the spatial and temporal dynamics of bacterial effector proteins within the host cellular environment.

Bacterial pathogens have a limited capacity to delivery bacterial toxins and effector proteins into host cells. The type III secretion system, for example, is thought to translocate between 20 and 50 effector molecules per second, which would result in low picomolar host cellular concentrations^8^. This situation poses biophysical problems for the pathogen as enzymes operating at low molecular concentrations can exhibit extreme fluctuations in reaction rates caused by natural variation in host cell size, morphology, and substrate availability^9^. Thus, the low concentrations of effector proteins, in the absence of highly localized signaling mechanisms, would result in unintended and deleterious phenotypic outcomes^10^. However, it remains unclear how the majority of effector proteins amplify their enzymatic activity within defined subcellular compartments of host cells.

The regulated targeting of proteins to the plasma membrane and other membrane-bound organelles is a key-defining feature of many eukaryotic signaling networks^11, 12^. In fact, several aspects of host membrane architecture make it a critical site for protein accumulation and a hub for local signal amplification. First, the ability of lipids to recruit cytosolic proteins onto a two-dimensional membrane surface has a powerful concentration effect within the cell. Second, protein movement within membranes is much slower than in the cytoplasm, providing a physical barrier to protein diffusion. Third, certain lipid-types can be geographically restricted within the cell, building spatially defined membrane microdomains that generate selectivity in many signal transduction systems. Finally, membrane surfaces are often used as physical scaffolds for the assembly of multi-protein complexes that display robust detection, amplification, and decoding of input signals. In these contexts, it seems a reasonable assumption that bacterial effector protein acquisition of lipid binding domains would offer a simple, yet flexible, strategy for bacterial pathogens to locally amplify and coordinate host signal transduction systems, in both space and time, during the course of infection.

Here, we combined a gain-of-function genetic screen in yeast with fluorescence microscopy to systemically interrogate bacterial effector protein and host membrane interactions. This integrative approach revealed that ~30% of the bacterial effector protein repertoire tested associates with membranes of eukaryotic cells. Further characterization of phospholipid-binding interactions revealed specific membrane-targeting features of several effectors including *Shigella* IpgB1, which spatially localizes Rac1 activation during bacterial invasion. This work provides a resource and experimental methodology to examine the spatiotemporal function of effector proteins in the host cellular environment.

## Results

### Identification of effector proteins that interact with yeast membranes

To generate a library of bacterial effector genes, we curated the literature for confirmed type III secreted effector proteins encoded in the SPI-I and SPI-2 genetic loci of *S almonella enterica* serovar Typhimurium (*St*), the Hrp pathogenicity island of *Pseudomonas syringae* (*Ps*), the pWR100 virulence plasmid of *Shigella flexneri* (*Sf*), and the locus of enterocyte effacement (LEE) as well as the nine other effector loci (non-LEE) of Enterhaemorrhagic *E. coli* O157:H7 (*Ec*). In addition, the library was expanded to include the type IV secreted effectors from the Icm/Dot and VirB/D4 systems from *Legionella pneumophila* (*Lp*) and *Bartonella henselae* (*Bh*), respectively. In total, 200 bacterial effector genes were cloned into the GATEWAY compatible pENTR/D vector to facilitate rapid transfer of genes into a wide array of yeast, mammalian, and bacterial expression vectors (Supplementary Table 1).

**Fig. 1:**
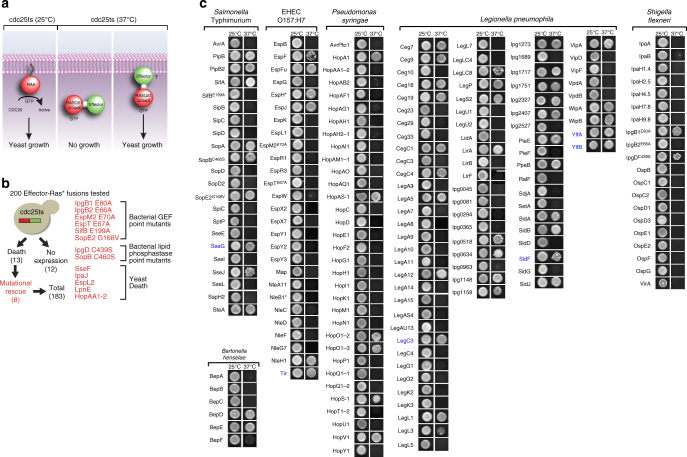
The Ras-rescue screen. **a** Schematic diagram of the temperature-sensitive Ras-rescue system used to identify membrane-associated effectors in yeast. **b** Experimental setup for construction of the Ras*-effector fusion library and mutants that alleviated yeast growth inhibition. Ras*-effector-HA fusion proteins (labeled *red-green*) were expressed in cdc25ts and assayed for growth inhibition. Catalytically dead mutations (if known) were made in each effector that caused yeast growth defects, allowing for those effectors to be included in the screen. The catalytic residues for EspL2^*Ec*^, LpnE^*Lp*^, SseF^*St*^, IpaJ^*Sf*^, and HopAA1-2^*Ps*^ were unknown and, therefore, these effectors could not be included in the screen. **c** Growth assay for the cdc25ts yeast strain harboring the indicated effector genes grown at the non-restrictive temperature (25 °C) or the restrictive temperature (37 °C). Effectors that allow for yeast growth at the restrictive temperature reconstituted Ras* localization to the membrane. Effectors known to have transmembrane domains are *colored* in *blue* as confirmation of the screen

Next, we adapted a “Ras-rescue screen” to identify bacterial effector proteins that interact with intracellular membranes of the yeast *Saccharomyces cerevisiae* as a model organism (Fig. 1a)^13^. This screen is based on the requirement of RAS GTPase, an essential gene that promotes cell growth and division, to interact with cellular membranes for proper signal propagation. It is known that RAS is targeted to the plasma membrane via fatty acid modification of the C-terminal CaaX sequence and membrane-associated RAS is directly activated by the guanine nucleotide exchange factor CDC25^13^. A yeast strain with a temperature-sensitive allele for CDC25 (cdc25ts) grows normally at the permissive temperature of 25 °C, but fails to grow at 37 °C as cdc25ts fails to activate endogenous RAS. The growth defect of cdc25ts at 37 °C could be rescued by heterologous expression of a constitutively active (Q61L), non-farnesylated (∆CaaX) RAS protein (herein referred to as Ras*) that is fused to a membrane-targeting domain (Fig. 1a)^13^. In essence, if a protein of interest drives Ras* to a cellular membrane, it will promote cdc25ts yeast growth at the non-permissible temperature (37 °C) (Fig. 1a). Because RAS has been shown to signal from the plasma membrane as well as membrane-bound organelles^14, 15^, we reasoned that Ras* fusions to bacterial effector proteins that target host membrane systems would rescue cdc25ts yeast growth at 37 °C (Fig. 1a).

All 200 bacterial effector genes were fused in-frame to the C terminus of Ras* and transformed into cdc25ts yeast. We were unable to recover colonies transformed with 13 bacterial effector genes (encoding IpgB1^*Sf*^, IpgB2^*Sf*^, IpgD^*Sf*^, IpaJ^*Sf*^, EspM^*Ec*^, EspL2^*Ec*^, EspT^*Ec*^, SifB^*St*^, SopE2^*St*^, SopB^*St*^, SseF^*St*^, LpnE^*Lp*^, and HopAA1-2^*Ps*^), suggesting that these proteins caused a growth arrest phenotype in yeast (Fig. 1b). Indeed, inactivating point mutations in the bacterial GEF domain of IpgB1^*Sf*^, IpgB2^*Sf*^, EspM^*Ec*^, EspT^*Ec*^, SifB^*St*^, and SopE2^*St*^; and the catalytic phosphatase domain of IpgD^*Sf*^ and SopB^*St*^ resulted in successful yeast transformation (Fig. 1b). We confirmed the expression of 190 full-length Ras*-effector protein fusions in the cdc25ts mutant at the permissible growth temperature of 25 °C.

**Fig. 2:**
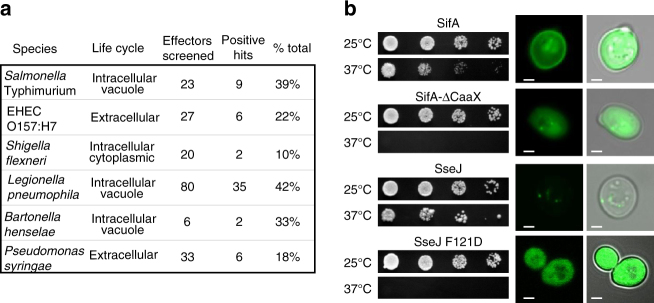
Summary and validation of the Ras-rescue screen. **a** Table of results summarizing the pathogens studied, effectors screened, and percentage of effector repertoire from each pathogen that localized to eukaryotic membranes. **b** (*Left*) Ras-rescue screen of wild-type SifA (SifA, a SifA-ΔCaaX (mutation that removes the CaaX box from the C terminus of Sifa and no longer allows for prenylation), SseJ, or SseJ F121D (mutation that blocks binding of SseJ to membrane-bound RhoA). (*Right*) Micrographs of EGFP-tagged molecules with bright field overlay on the right. *Scale bar* is 1 μm

From a total of 190 Ras*-effector proteins validated, 60 promoted yeast growth at 37 °C (Figs. 1c and 2a). This value was surprisingly large as yeast is not a natural host for these pathogens, which infect animal and plant cells. However, the lipid composition and membrane-binding components are highly conserved from yeast to humans^16^. Indeed, several bacterial effector proteins that rescued cdc25ts growth defects encoded transmembrane (TM) spanning domains (e.g., Tir^*Ec*^, SidF^*Lp*^, YlfA^*Lp*^, YlfB^*Lp*^, and SseG^*St*^) (Fig. 1c), were post-translationally modified by mammalian prenyltransferases (e.g., SifA^*St*^), or interacted with mammalian peripheral membrane-binding proteins (e.g., SseJ^*St*^) (Fig. 2b)^17, 18^. In confirmation that these mechanisms are conserved in yeast, point mutations in the CaaX box of SifA^*St*^ that abolished its site of prenylation^17^ or mutations in SseJ^*St*^ that inhibits its known interaction with membrane-bound RhoA GTPase^18^, failed to rescue cdc25ts yeast growth at 37 °C and disrupted yeast membrane localization (Fig. 2b). These experiments show that yeast can be used as a model organism to identify host pathways required for host membrane targeting of bacterial effectors, and provided additional validation of the membrane interactions identified in the Ras screen.

A potential caveat of this screen was that fusion of Ras* to the amino terminus of bacterial effector proteins may cause mis-localization through disruption of critical localization or retention sequences required for proper membrane interactions. For example, the *Pseudomonas syringae* effectors HopF2 and AvrPto1 are targeted to plant membranes via amino-terminal myristoylation^19, 20^ and the *Salmonella* Typhimurium effectors SseI and SspH2 localize to the *Salmonella*-containing vacuole (SCV) in human cells via amino-terminal palmitoylation^21^. Given that the Ras* fusion protein would mask the fatty acid acceptor sites, it is not surprising that these known membrane-associated bacterial effector proteins did not rescue cdc25ts growth at 37 °C (Fig. 1c). It is therefore clear that the Ras-rescue screen can result in false-negative assumptions. Nevertheless, we have identified 40 previously unreported membrane-binding interactions and also confirmed the membrane localization of previously uncharacterized bacterial effector proteins (Supplementary Table 2).

### Functional evaluation of membrane-binding effectors

It is interesting to note that each of the bacterial pathogens tested possessed at least one effector protein that interacted with yeast cellular membranes (Fig. 2a). However, effector proteins from *S.* typhimurium and *L. pneumophila* represented 78% of the total positive clones (Fig. 2a). This enrichment may be due to the intracellular, vacuolar lifestyles of these organisms, and the role of bacterial effector proteins to alter the dynamics of host vesicle trafficking systems during infection. Although this idea is speculative, it was clear that bacterial effectors identified in the Ras-rescue screen span a wide range of biological functions including signal transduction, vesicle trafficking, cytoskeletal dynamics, and host immune regulation (Supplementary Table 2). In addition, literature inquiries indicated that positive hits in the Ras-rescue screen displayed a wide variety of enzymatic activities (Supplementary Table 2). We identified three lipid phosphatases (SopB^*St*^, IpgD^*Sf*^, and SidF^*Lp*^), three Rho-family GEFs (SopE2^*St*^, SifA^*St*^, and IpgB1^*Sf*^), three actin nucleation factors (VipA^*Lp*^, EspF^*Ec*^, and EspFu^*Ec*^), an E3 ubiquitin ligase (SopA^*St*^), a cholesterol esterase (SseJ^*St*^), a protein kinase (NleH^*Ec*^), a phospholipase (CegC1^*Lp*^), and a sphingosine-1 phosphate lyase (LegS2^*Lp*^). These findings suggest that the evolution of membrane-targeting domains is a common strategy that may be used to enhance the signaling specificity and/or efficacy of effector proteins.

### Subcellular localization of bacterial effector proteins in yeast

**Fig. 3:**
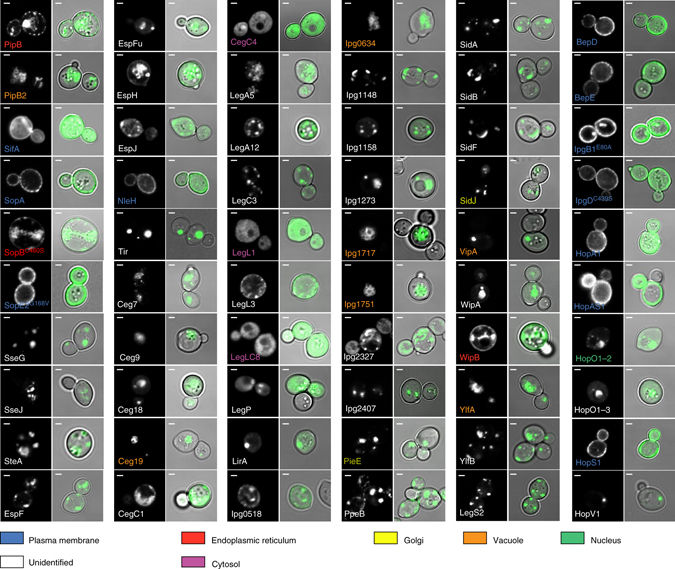
Bacterial effector protein localization in yeast. Subcellular localization of EGFP-tagged membrane-interacting effectors in yeast. *Scale bar* is 1 μm

To confirm the Ras-rescue screen, EGFP protein was fused to the amino terminus of all 60 bacterial effector genes and subcellular localization was monitored by fluorescence microscopy in yeast. We found 11 bacterial effector proteins (out of 60) localized to the plasma membrane of yeast, including *Salmonella* SifA, SopA, SopE2, EHEC O157:H7 NleH, and *Bartonella* BepD and BepE, *Shigella* IpgB1 and IpgD, and *P. syringae* HopA1, HopAS1, and HopS1 (Fig. 3). In addition, we found that bacterial effector proteins were targeted to the nuclear membrane (HopO1-2^*Ps*^), endoplasmic reticulum (PipB^*St*^, SopB^*St*^, and WipB^*Lp*^), Golgi apparatus (PieE^*Lp*^ and SidJ^*Lp*^), and yeast vacuole (PipB2^*St*^, Ceg19^*Lp*^, lpg0634^*Lp*^, lpg1717^*Lp*^, lpg1751^*Lp*^, YlfA^*Lp*^, and VipA^*Lp*^) (Fig. 3). These data are consistent with the ability of RAS to signal from plasma membrane as well as from multiple organelle sites^15^. Finally, 37 effector proteins accumulated in one or more subcellular structures, however the small size of yeast (~2 µm) and the transient nature of membrane trafficking systems limited our ability to define their precise location by fluorescent microscopy. The localization in mammalian cells has been previously reported for several bacterial effectors whose sites of localization we were unable to precisely define in yeast: SseG^*St*^, SseJ^*St*^, SteA^*St*^, EspF^*Ec*^, EspH^*Ec*^, EspJ^*Ec*^, Tir^*Ec*^, Ceg9^*Lp*^, CegC3^*Lp*^, SidF^*Lp*^, YlfB/LegC2^*Lp*^, and LegS2^*Lp*^ (Supplementary Table 2). These data suggest that yeast can be used as a tool to identify molecular mechanisms of effector localization. Surprisingly, only three proteins, CegC4^*Lp*^, LegL1^*Lp*^, and LegLC8^*Lp*^, showed featureless and diffuse localization patterns in yeast. These either represent false-positive interactions or suggest that their membrane-binding partner may be readily saturable (e.g., protein:protein interaction).

It is important to note that for some effectors (including PipB2^*St*^, SifA^*St*^, SopA^*St*^, SopB^*St*^, PieE^*Lp*^, YlfA/LegC7^*Lp*^, and BepE^*Bh*^) the localization in yeast does not precisely match the localization previously reported in mammalian cells (Supplementary Table 2). However, the molecular mechanisms of effector targeting are likely conserved from yeast to human. For example, SifA is localized to the *Salmonella*-containing vacuole during infection and to the plasma membrane in yeast. Nevertheless, both targeting mechanisms require prenylation of the C-terminal CaaX box (Fig. 2b).

### Study of uncharacterized *Legionella* effector proteins

**Fig. 4:**
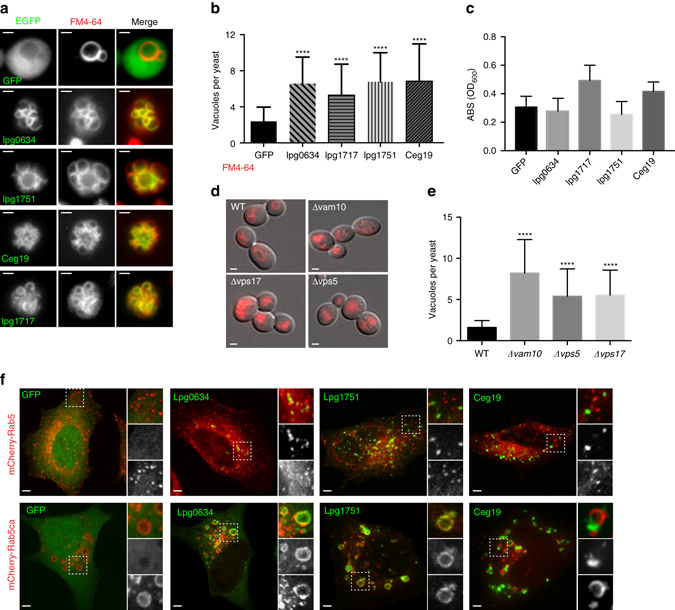
*Legionella* effector proteins target endocytic compartments in eukaryotic cells. **a** Fm4-64 dye (*red*) labeled vacuoles in *S. cerevisiae* expressing the *Legionella* effectors, GFP-Lpg0634, GFP-Lpg1717, GFP-Lpg1751, GFP-Ceg19, or GFP control. *Scale bar* is 1 µm. **b** Average number of vacuoles per yeast cell expressing either EGFP, the *Legionella* effectors (Lpg0634, Lpg1717, Lpg1751, or Ceg19). The data are displayed as mean ± SD for the indicated number of yeast cells from three independent experiments. Average number of vacuoles per wild-type yeast cells is between 1 and 3. Dunnett’s multiple comparisons test used for statistical analysis (95% CI, ****p < 0.0001). **c** Growth assay of yeast expressing EGFP or EGFP-tagged *Legionella* effectors from (**a**) after 72 h induction (**d**) FM4-64 labeled vacuoles in yeast strains with specified Class B vacuole fragmentation gene deleted. *Scale bar* is 1 µm. **e** Average number of vacuoles per yeast cell seen in WT or indicated mutants. The data are displayed as mean ± SD for the indicated number of yeast cells from three independent experiments. Dunnett’s multiple comparisons test used for statistical analysis (95% CI, ****p < 0.0001). **f** Fluorescence microscopy of HeLa cells co-transfected with the *Legionella* effector indicated (*green*) and either wild-type Rab5 or constitutively active Rab5 (*red*). The *boxed* region is magnified × 2 and the *top* is the merge, the *middle* the GFP signal, and the *bottom* the mCherry signal. *Scale bar* is 5 µm

We expect these results to be useful to advance two areas of research: (1) the biological function of uncharacterized bacterial effector proteins, and (2) the molecular mechanism of their subcellular distribution. To address the former, we initially sought to identify bacterial effector proteins that regulate vesicle trafficking and membrane fusion of the yeast vacuole, an organelle that is easily visualized by both light and fluorescence microscopy and is evolutionarily related to the mammalian endolysomal trafficking system. Using FM4-64 dye to label the vacuole and by monitoring EGFP-tagged effector protein localization, we identified four *Legionella* proteins that specifically associated with the yeast vacuolar membrane (Fig. 4a). Several lines of evidence suggested that Lpg0634^*Lp*^, Lpg1717^*Lp*^, Lpg1751^*Lp*^, and Ceg19^*Lp*^ regulate membrane trafficking events from these sites. First, ectopic expression of each bacterial effector protein induced a multiple-vacuole phenotype characterized by an increase in organelle number from an average of two vacuoles per wild-type yeast cell to an average of six vacuoles in bacterial effector-expressing yeast cells (Fig. 4b). Second, Lpg0634^*Lp*^, Lpg1751^*Lp*^, and Ceg19^*Lp*^ accumulated at the boundaries of docked vacuoles (Fig. 4a), which is a specific site of membrane fusion^22^. Third, the *Legionella* effector proteins induced a class B vacuole fragmentation phenotype similar to those caused by loss-of-function mutations in components of the yeast vacuole fusion machinery, including Vam10, Vps5, and Vps17 (Fig. 4d, e)^23^. Lastly, the multivacuole phenotype is probably not caused by general stress induced by the expression of the *Legionella* effectors, as there was no change in yeast growth in GFP-Lpg0634^*Lp*^, GFP-Lpg1717^*Lp*^, GFP-Lpg1751^*Lp*^, and GFP-Ceg19^*Lp*^-expressing yeast compared to GFP control (Fig. 4c).

Similar to results found in yeast, EGFP-tagged Lpg0634^*Lp*^, Lpg1751^*Lp*^, and Ceg19^*Lp*^ expressed in HeLa cells were found to localize in vesicular patterns reminiscent of endomembrane trafficking organelles, but did not co-localize strictly with any single subcellular marker (Fig. 4f). As the mammalian secretory pathway comprises numerous membrane sub-systems, we co-expressed EGFP-tagged bacterial effector proteins with a constitutively active Rab5 mutant that collapses endocytic membranes into large membrane compartment. If indeed the *Legionella* effector associated with endoctyic vesicles, it should co-localize with ca-Rab5 under these conditions. As shown in Fig. 4f, expression of ca-Rab5 induced the fusion of membrane vesicles harboring Lpg0634^*Lp*^ and Lpg1751^*Lp*^. Interestingly, Ceg19^*Lp*^-positive vesicles docked, but failed to fuse with the early endosomes when co-expressed with ca-Rab5 (Fig. 4e). These results support that the subcellular localization of bacterial effector proteins in yeast can be used to guide our analyses in higher eukaryotic organisms. We speculate that Lpg0634^*Lp*^, Lpg1717^*Lp*^, Lpg1751^*Lp*^, and Ceg19^*Lp*^ might regulate membrane fusion and fission events that promote the integrity of the *Legionella*-containing vacuole (LCV) in infected host cells.

### Loss-of-function PI-kinase screen identifies lipid-binding domains

**Fig. 5:**
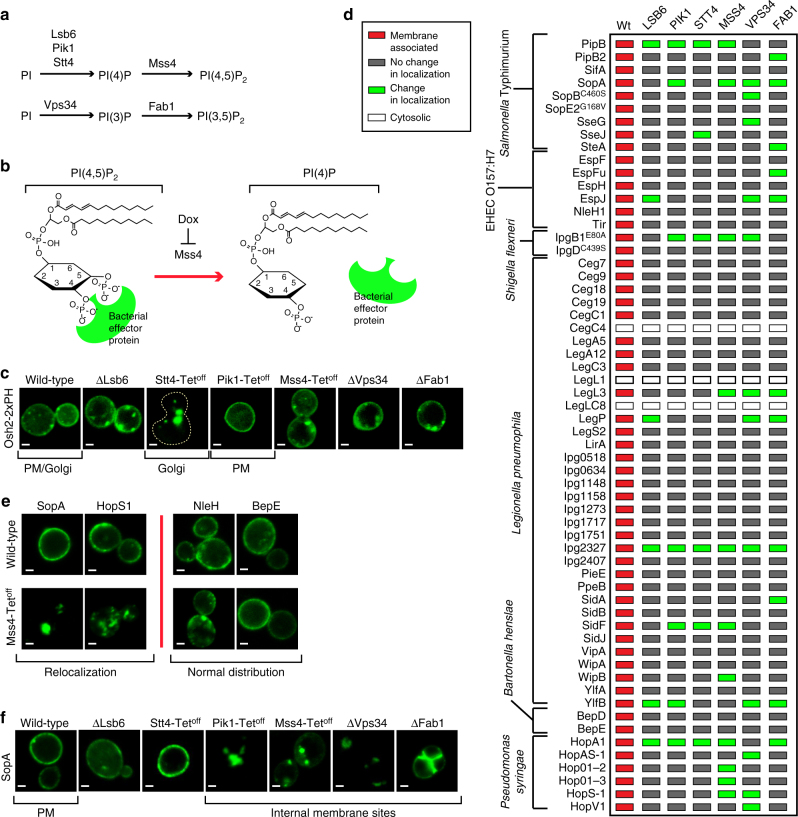
Membrane targeting of effectors in yeast with altered phosphoinositide levels. **a** PIP synthesis is regulated by six PI-kinases in yeast by the indicated pathways. **b** Design of the PI kinase screen. Genetic deletion or doxycycline repression of each PI kinase (Mss4 shown) depletes the PIP, causing loss of localization of effectors that have a membrane localization governed by PI(4,5)P_2_. **c** Localization of EGFP-Osh2 (a known PI4P binding protein) in wild-type *S. cerevisiae* cells and the six PI kinase yeast strains. **d** Graphical representation of all the membrane-associated effectors localization upon depletion of each of the six PI-kinases compared to localization in wild-type yeast (Supplementary Fig. 1). **e** Representative images of plasma membrane-localized effectors that were redistributed when MSS4 was repressed by the presence of doxycycline (example: SopA^*St*^ and HopS1^*Ps*^), or did not redistribute with MSS4 repression (example: NleH^*Ec*^ and BepE^*Bh*^). *Scale bar* is 1 μm. **f** Fluorescent images of GFP-fused SopA^St^ in wild-type *S. cerevisiae* cells and the six PI kinase yeast strains. *Scale bar* is 1 μm

We then sought to develop a methodology to identify yeast components guiding membrane targeting of each of the 57 effector proteins identified in the Ras-rescue screen. To this end, we focused on the evolutionarily conserved inositol phospholipid signaling system. The phosphorylation of phosphatidylinositide (PI) by isoform-specific PI kinases has a fundamental role in regulating the membrane–cytosolic interface in yeast, plants, and animal cells. Yeast cells have six PI kinases that phosphorylate the 3′, 4′, or 5′ positions of PI, generating a class of negatively charged inositol-containing phospholipids (known as PIPs) (Fig. 5a). The geographical restriction of specific PIPs within the cell, and the unique phosphorylation patterns on PIPs, act as site-specific signals on membranes that recruit proteins for the assembly of spatially localized functional complexes^24, 25^. Thus, inactivation of yeast PI-kinase genes would, in theory, cause mis-localization of EGFP-tagged bacterial effector proteins that require phospholipid interactions for membrane targeting, while providing a visual readout to identify novel modes of membrane association (Fig. 5b).

The expression of each yeast PI-kinase gene was inhibited by either isogenic knockout of the non-essential PI-kinase genes (VPS34, FAB1, and LSB6)^26^ or by doxycycline-mediated (Dox) repression of TetO_7_-promoter alleles of essential PI-kinase genes (PIK1, STT4, and MSS4)^27^. To confirm that repression of TetO_7_-promoter alleles is sufficient to disrupt specific PIP isoforms, we monitored the distribution of the PI4P-specific binding protein Osh2^28^, which shuttles between the plasma membrane and Golgi apparatus upon depletion of the essential PI4-kinases PIK1 and STT4, respectively (Fig. 5c)^29^. Consistent with previous findings, Dox inhibition of PIK1 and STT4 expression caused the redistribution of Osh2 to the PM or Golgi, respectively. Next, 57 EGFP-tagged bacterial effector genes were transformed into the PI-kinase-mutant strains in a one-to-one format, generating a mini-array of 342 potential host-pathogen interactions (Fig. 5d). The subcellular localization of EGFP-effector proteins were visualized by fluorescence microscopy and subsequently compared to their location in wild-type yeast (Fig. 5d and Supplementary Fig. 1).

As shown in Fig. 5d, 23 of 57 bacterial effector proteins were differentially localized when expressed in one or more of the PI-kinase-mutant strains. The majority of the effector proteins did not simply accumulate in the cytoplasm, but rather redistributed from their primary sites of localization to new subcellular compartments. For example, SopA^*St*^ and HopS1^*Ps*^ were redirected from the PM to internal sites by repression of Mss4, a PI-5 kinase that generates the major PM phospholipid PI(4,5)P_2_ (Fig. 5e). Depletion of PI(4,5)P_2_ did not simply disrupt plasma membrane integrity, as the peripheral localization of several bacterial effector proteins were unaltered under these conditions (Fig. 5e). Further examination revealed that *Salmonella* SopA relocated from the plasma membrane to internal punctate via loss of several PI kinases including PIK1, MSS4, VPS34, and FAB1, but not LSB6 or STT4 (Fig. 5f). We concluded that the sensitivity of bacterial effector proteins to the loss of multiple PI-kinase genes likely reflects bacterial effector protein affinity for several phospholipid isoforms. As manipulation of PI kinases will have effects on other membrane-associated proteins and processes, it is also possible that the mis-localization of EGFP-tagged bacterial effectors in the yeast PI-kinase deletion strains results from alterations in other PIP-dependent processes. Thus, biochemical analysis of effector and host PIP interactions are needed to confirm these results.

### Analyses of bacterial effector protein and host acidic phospholipid interactions

**Fig. 6:**
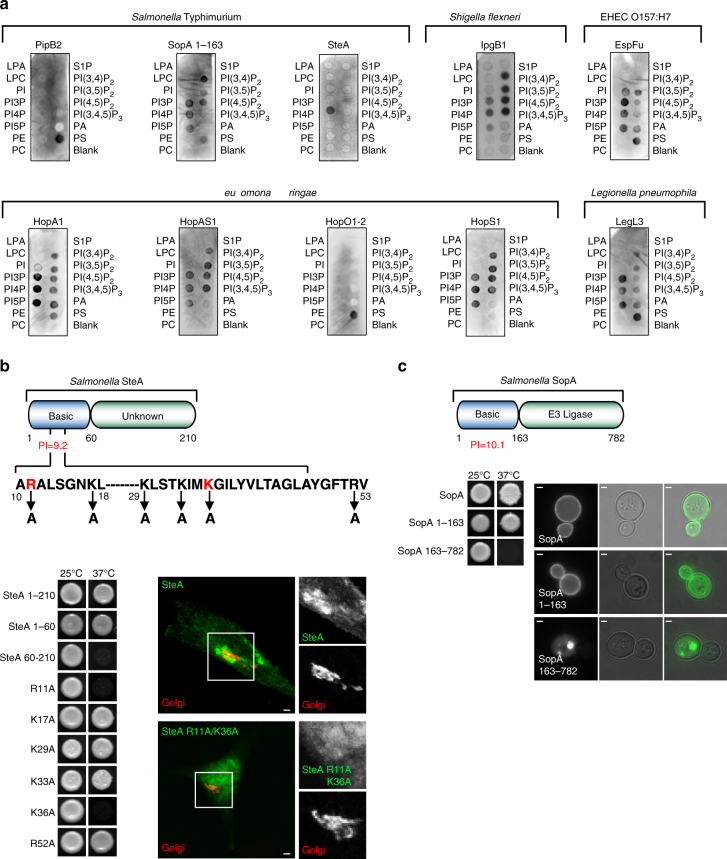
Direct phospholipid interactions of bacterial effector proteins. **a** PIP strips of the ten effectors that changed localization in the PI kinase screen, but were not predicted to have TM domains. **b** SteA^*St*^ truncations and mutants as listed were fused to the C terminus of Ras* and assayed for growth at the non-permissible temperature. Fluorescent microscopy of wild-type SteA^*St*^ or SteA^R11AK36A^ co-expressed with Golgi apparatus marker in HeLa cells (SteA: *green*, GM130: *red*). *Scale bar* is 5 μm and inset is ×4 magnified to the right. **c** Ras-rescue screen using the full-length, N terminus (1-163), and C terminus (163-end) of SopA^*St*^. Fluorescent micrographs of yeast expressing GFP fusions with full-length, the N terminus, or the C terminus of SopA^*St*^. *Scale bar* is 1 μm

To determine whether the effector proteins that exhibited PIP-dependent membrane localizations were able to bind directly to PIPs, we performed in vitro interaction studies between the 23 bacterial effector proteins identified in the loss-of-function PI-kinase screen and a panel of acidic phospholipids in vitro. Unfortunately, laboratory *E. coli* failed to express full-length bacterial effector proteins, likely due to the absence of pathogen-specific chaperones that are required for their solubility and stability. To overcome this problem, each bacterial effector protein was tagged with a tandem 8 amino acid “Strep-tag” and expressed and purified from HEK293T cell lysates by Strep-Tactin affinity chromatography. Purified proteins were overlaid onto nitrocellulose membranes spotted with a variety lipid species (PIP strips) and assessed for binding by anti-Strep-tag immunoblot. Seven effector proteins had no interaction with any lipid tested and we reasoned either the recombinant proteins were misfolded during purification or these bacterial effector proteins associate indirectly to endogenous PIP-binding proteins in yeast. In addition, we found that six effector proteins (SseG^St^, Ceg18^*Lp*^, PpeB^*Lp*^, SidA^*Lp*^, SidF^*Lp*^, and YlfB^*Lp*^) could not be assayed under these conditions, as they produced significant background binding (likely due to the hydrophobic nature of predicted transmembrane spanning sequences). However, 10 bacterial effector proteins had affinity for anionic phospholipids in vitro (Fig. 6a). A few effector proteins bound a single phospholipid, whereas the remainder associated with multiple lipid forms. This is consistent with two different modes of lipid binding: stereoselective and general charge sensing^25^. It is also important to note that the N terminus of HopA1^*Ps*^ is homologous to the *Vibrio paraheamolyticus* effector VopR that was recently shown to use PIPs as folding substrate upon translocation into eukaryotic cells^30^. Thus, the loss-of-function screening in yeast can be used to identify molecular mechanisms of bacterial effector protein binding to eukaryotic membranes.

**Fig. 7:**
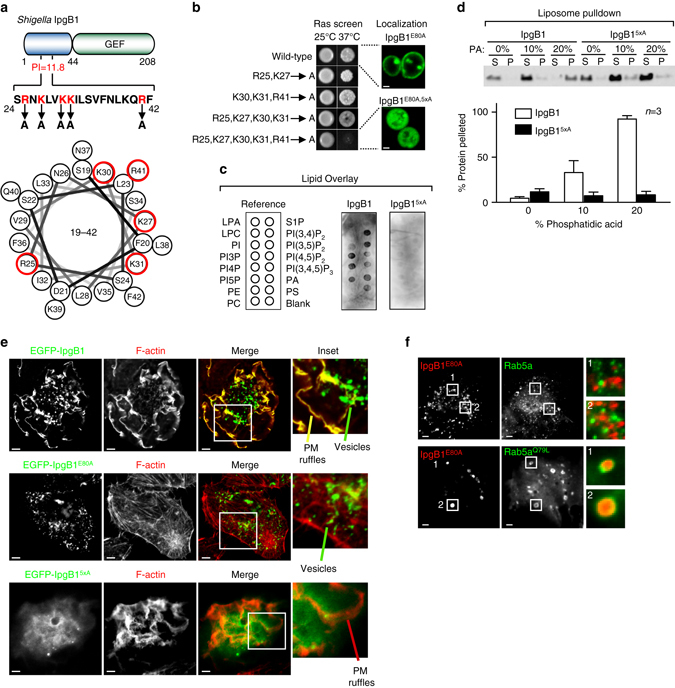
IpgB1 polybasic domain confers phospholipid binding and membrane targeting. **a** Cartoon diagram of the IpgB1 protein depicting the C-terminal GEF domain and N-terminal polybasic domain. In addition, a helical wheel projection of amino acids 19–42 is shown below with amino acids targeted for mutagenesis highlighted in *red*. **b** Images of *cdc25ts* yeast expressing IpgB1^E80A^ or the indicated mutants on an IpgB1^E80A^ mutant background fused to a soluble constitutively active Ras. Membrane localization confers yeast growth at 37 °C. EGFP-IpgB1^E80A^ or EGFP-IpgB1^E80A,5xA^ is shown to the right. *Scale bar* is 1 μm. **c** Lipid overlay assays using recombinant, Strep-affinity purified IpgB1 or IpgB1^5xA^ (R25A K27A K30A K31A R41A). Membranes are depicted in the same orientation as the cartoon on the left. **d** Liposome sedimentation studies using recombinant IpgB1 or IpgB1^5xA^ protein. (*Top*) Western blot analysis looking at protein levels in the supernatant (S) or pellet (P) of sedimented membranes containing the indicated molar % of phosphatidic acid. Full blot is shown in Supplementary Fig. 3. (*Bottom*) Quantification of the relative percentage of IpgB1 or IpgB1^5xA^ within the pellet. Mean ± standard deviation is plotted for three independent experiments. **e** Fluorescent micrographs of HeLa cells expressing EGFP-tagged (*green*) IpgB1, IpgB1^E80A^ (Rac1 binding mutant), or IpgB1^5xA^ stained with Alexa Fluor 594 Phalloidin (*red*) to mark the actin. *Scale bar* is 5 μm and insets are ×2 magnified. IpgB1 localization on the plasma membrane ruffles and on endocytic vesicles are marked. *Scale bar* is 5 µm. **f** Fluorescent micrograph of HeLa cells expressing mCherry-tagged IpgB1^E80A^ (*red*) and either EGFP-Rab5 or EGFP-Rab5a^Q79L^. mCherry-IpgB1 becomes enriched within the enlarged endosomes generated in cells expressing the constitutively active Rab5a^Q79L^ mutant. *Scale bar* is 5 μm and insets are × 5 magnified

We next sought to identify specific protein signatures that may drive lipid-dependent subcellular localization. Neither sequence- nor structural-based bioinformatics (e.g., PH, PHOX, FYVE, etc.) revealed canonical lipid-binding domains in the bacterial effectors analyzed. However, three bacterial effector proteins (SteA^*St*^, SopA^*St*^, and IpgB1^*Sf*^) possessed short sequence motifs enriched in basic and aromatic residues that exhibited isoelectric points greater than 8.5, suggesting that they are positively charged at physiological pH (Figs. 6b, c and 7a). These properties are often found in domains that bind negatively charged phospholipids^31^. Truncation analysis confirmed that the NH_2_-terminal 60 amino acids of SteA^*St*^ and the NH_2_-terminal 163 amino acids of SopA^St^ were necessary and sufficient for membrane localization in yeast (Fig. 6b, c). In addition, mutations in the basic residues R11/K36 disrupted the previously described Golgi localization of SteA^*St*^ in mammalian cells (Fig. 6b)^32^.

### *Shigella* IpgB1 targets acidic phospholipids via N-terminal polybasic region

Out of the 10 effector proteins found to bind phospholipids, the *Shigella* IpgB1 was one of the few with known enzymatic activity, thus providing a tractable model to determine how membrane targeting might control the spatial and temporal activity of effector proteins. IpgB1 belongs to the WxxxE/SopE family of bacterial guanine-nucleotide exchange factors (GEFs)^33, 34^. The defining feature of this family is a conserved 180 amino acid GEF domain that converts GDP-bound rho-family GTPases to their GTP-bound active form^34, 35^. IpgB1 promotes *Shigella* invasion into non-phagocytic cells through the activation of Rac1, and to a lesser extent Cdc42, signaling cascades^34, 36^. In agreement with our yeast localization data (Fig. 3), previous studies have also shown that IpgB1 is targeted to host plasma membrane, yet the mechanism and biological function of this interaction has not yet been resolved^37, 38^.

Previous studies demonstrated that the N-terminal region of IpgB1 includes a chaperone-binding domain that is necessary for interactions with Spa15 in *Shigella* ^37^ and that the C-terminal 180 residues contain the GEF domain that binds and activates Rac1 GTPase (Fig. 6a)^34^. Because inactivating mutations in the GEF domain (IpgB1^E80A^) that prevent Rac1 binding and activation had no effect on plasma membrane localization in yeast (Fig. 1c), it was reasonable to seek membrane-binding features present in the N terminus. Secondary structural predictions suggest that residues 19–42 form an amphipathic helix that is enriched in basic and hydrophobic amino acids (Fig. 7a). This region is predicted to exhibit a PI >11, suggesting that it is positively charged under physiological conditions. To determine whether the amphipathic helix mediates protein–lipid interactions, we first took advantage of the Ras-rescue screen in yeast due to its high sensitivity for detecting protein–membrane interactions. Arginine and lysine residues were selectively mutated as these residues would provide the positive charge required for acidic phospholipid interaction. No single mutation conferred loss of membrane-binding functions (Fig. 7b). However, a combinatorial mutation of five basic residues (R25, K27, K30, K31, R41) to alanine (herein referred to as IgpB1^5xA^) abolished membrane-based RAS* signaling (Fig. 7b). We confirmed these results by comparing the localization of catalytically inactive IpgB1^E80A^ to the lipid-binding mutant IpgB1^5xA–E80A^ in yeast. GFP-IpgB1^E80A^ localized to the plasma membrane, whereas the GFP-IpgB1^E80A,5xA^ exhibited diffuse localization (Fig. 7b). To determine whether the amphipathic helix directly mediates protein–lipid interactions, we purified full-length, STREP-tagged IpgB1^5XA^ from mammalian cells and overlaid the protein onto PIP strips. In contrast to wild-type IpgB1, IpgB1^5xA^ had only background levels of PIP binding (Fig. 7c). Finally, we performed liposome sedimentation assays to determine whether the interaction between IpgB1 and acidic phospholipids can be reconstituted on a membrane bilayer using purified components. Only background levels of wild-type IpgB1 sedimented with membranes composed of 100% phosphatidyl choline (PC) (Fig. 7d). In contrast, vesicles containing 20% phosphatidic acid (PA) sedimented the majority of IpgB1 protein, but failed to interact with IpgB1^5xA^ mutant protein (Fig. 7d). Taken together, these studies suggest that IpgB1 utilizes an amphipathic helix enriched with basic residues to interact directly with acidic phospholipids.

Next, we sought to understand how the amphipathic helix controls the location of IpgB1 signaling in mammalian cells expressing EGFP-tagged IpgB1. Consistent with previous reports, EGFP-tagged IpgB1 localized to actin-rich plasma membrane ruffles (Fig. 7e)^31^. We also observed IpgB1 enriched on intracellular vesicles(Fig. 7e). To confirm the endocytic nature of the IpgB1 vesicles, we induced endocytic vesicle fusion by overexpression of GTP-locked mutant of Rab5 (Q79L)^39^. In control experiments, wild-type Rab5a localized to early endocytic vesicles, yet did not perturb the endocytic network or IpgB1 vesicle localization (Fig. 7f). In contrast, IpgB1 colocalized extensively with the enlarged endosomes in cells expression of Rab5 (Q79L) indicating that the IpgB1-bound vesicles are functionally connected to the endocytic compartment (Fig. 7f). Having established the dual localization of IpgB1 to actin-rich membrane ruffles and endocytic vesicles, we then tested the requirements of the N-terminal lipid-binding domain and the C-terminal GEF domain for mediating membrane interactions and Rac1 activation. Importantly, the phospholipid-binding domain of IpgB1 was essential for both plasma membrane and endocytic localization as the catalytic mutant of IpgB1 (IpgB1^E80A^) localized to endocytic vesicles, whereas IpgB1^5xA–E80A^ mutant displayed featureless, cytoplasmic staining (Fig. 7e).

### Acidic phospholipids direct IpgB1 to sites of *Shigella* invasion

**Fig. 8:**
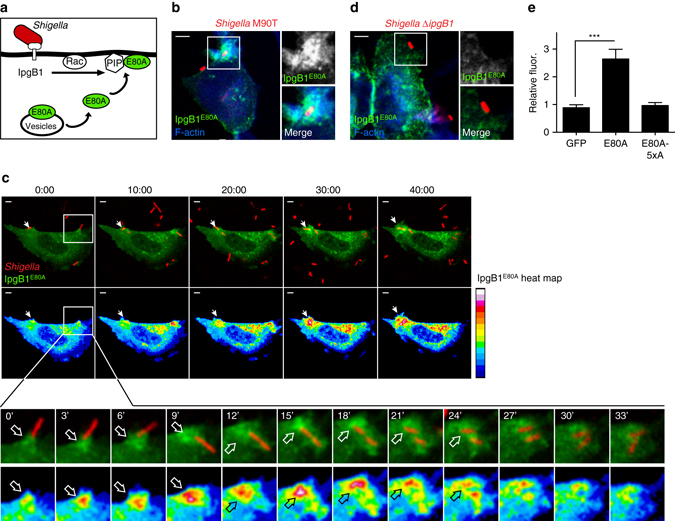
IpgB1 is recruited to site of *Shigella* invasion. **a** Cartoon diagram of the strategy to monitor the IpgB1 subcellular localization during *Shigella* infection. mCherry-expressing *Shigella* inject IpgB1 into host cells expressing the activity probe EGFP-IpgB1^E80A^. **b** Fluorescent micrograph of a HeLa cell expressing EGFP-IpgB1^E80A^ (*green*) and infected with mCherry-expressing wild-type *Shigella* M90T (*red*). AlexaFluor 350 Phalloidin is used to visualize the F-actin (*blue*) architecture. The *boxed* region contains the phagocytic cup and is in enlarged ×2 depicting the EGFP-IpgB1^E80A^ signal (*top*) and the merged image (*bottom*). *Scale bar* is 5 μm. **c** Time-lapse fluorescent microscopy of *Shigella*-mCherry (*red*) infecting an EGFP-IpgB1^E80A^ (*green*) expressing HeLa cell (*top*). Heat map of the pixel intensity of EGFP-IpgB1^E80A^ at the indicated time points. *Scale bar* for the heat map is shown to the *right* (*bottom*). *Scale bar* is 5 μm. **d** Fluorescent micrograph of a HeLa cell expressing EGFP-IpgB1^E80A^ (*green*) and infected with mCherry-expressing *ShigellaΔipgb1* (*red*). Figures are displayed as in (**b**). **e** Quantification of recruitment of lipid probes. Values reported as a fold increase of fluorescent signal in the phagocytic cup over the cytosol and are from five independent experiments

*Shigella flexneri* invades host cells through a trigger mechanism that requires the type III translocation of IpgB1 and subsequent activation of Rac1 at the sites of bacterial attachment^34, 36, 40^. We found that *Shigella* induces phospholipid accumulation at sites of invasion with high enrichment of PA, phoshpatidyl serine (PS), and phosphatidylinositol-3,4,5 phosphate (PI3,4,5P_3_) (Supplementary Fig. 2). We then attempted to monitor the subcellular localization of flag-tagged IpgB1 translocated during *Shigella* infection, yet this procedure failed perhaps due to the extremely low levels of type III protein secretion during infection. To then determine whether IpgB1 can be targeted to sites of *Shigella* infection via phosphoinositide accumulation, infection experiments were performed on HeLa cells ectopically expressing low levels of EGFP-IpgB1^E80A^. If phospholipid accumulation at sites of *Shigella* invasion drives IpgB1 to the plasma membrane, we predict that EGFP-IpgB1^E80A^ (a mutant that binds phosphoinositides but does not activate Rac1) would relocalize from endocytic vesicles to membrane ruffles produced by *Shigella* (Fig. 8a). Indeed, EGFP-IpgB1^E80A^ was recruited to the site of *Shigella* host cell interaction (Fig. 8b, c). Consistent with the notion that recruitment of EGFP-IpgB1 is triggered by cell invasion, the invasion-defective mutant *ΔipgB1* failed to recruit EGFP-IpgB1^E80A^ to sites of bacterial attachment (Fig. 8d). In addition, the recruitment of IpgB1^E80A^ is strictly dependent upon phospholipid interactions as IpgB1^5xA–E80A^ (Rac1 and phospholipid-binding mutant) was unable to be concentrated in phagocytic cups (Fig. 8e). Finally, time-lapse imaging revealed that the GFP-IpgB1^E80A^ probe rapidly accumulated in the phagocytic cup and was released from the plasma membrane shortly after bacterial internalization (Fig. 8c). Taken together, these data suggest that a polybasic region within the IpgB1 protein targets IpgB1 to the plasma membrane and endocytic vesicles within mammalian cells through the recognition of the host lipid bilayer.

## Discussion

Here, we present a systematic analysis of the subcellular localization of 190 bacterial type III and type IV secreted effector proteins from six bacterial pathogens in the yeast model organism. Results from this study argue that subcellular targeting is a common property of bacterial effectors and indicates that specific features of eukaryotic membranes may organize bacterial-based signaling systems in both space and time. Furthermore, we speculate that many bacterial effector proteins include as of yet unidentified interaction domains that provide additional regulation to ensure the efficient signaling capabilities of bacterial effectors following translocation into the host cell. In addition, we describe a direct interaction between IpgB1 and acidic phospholipids that is required for the previously described localization of IpgB1 within eukaryotic cells^37, 38^. Importantly, we show that the localization of this motif is dynamically regulated during *Shigella* invasion (Fig. 8c), suggesting a mechanism to spatially and temporally regulate effector protein function. Lastly, this work extends the power of yeast genetics to uncover host molecules that define the location of bacterial effector proteins in the eukaryotic cellular environment.

The RAS-rescue screen correctly identified several bacterial effectors with transmembrane, fatty acid, and protein:protein based localization mechanisms, illustrating the diversity of membrane interaction mechanisms that can be identified in yeast. In agreement with published studies in mammalian cells, we found expression of EGFP-fusions in yeast of NleH^*Ec*^, BepD^*Bh*^, IpgB1^*Sf*^, IpgD^*Sf*^ localized to the plasma membrane, PipB^*St*^ was found at the ER, and VipA^*Lp*^, Ceg19^*Lp*^ localized to the yeast vacuole, an organelle similar to the mammalian lysosome (Supplementary Table 2). In addition, our results suggest that many bacterial effector proteins contain cryptic membrane-targeting features. It is likely that additional studies on the newly identified localizations of WipB^*Lp*^ at the ER, SidJ^*Lp*^ to the Golgi, and Lpg0634^*Lp*^, Lpg1717^*Lp*^, and Lpg1751^*Lp*^ to the vacuole, will identify the domains that are responsible for the effector–membrane interactions in context of bacterial infection.

Prior to this study, only a small handful of bacterial effector proteins have been found to specifically bind acidic phospholipids^41^. SteA the *Salmonella* effector was recently shown to interact with PI4P through a N-terminal α-helix and in agreement with our data (Fig. 6a) this interaction was shown to be dependent on lysine 36 within this domain (Fig. 6b). Although the *Legionella* type IV effector DrrA/SidM was excluded from our screen because it did not express as a full-length fusion protein with RAS*, it does contain a COOH-terminal PI4P-binding site that does not share any sequence or structural homology to eukaryotic PIP-binding proteins. Thus, the membrane-targeting motifs of bacterial effector proteins, including the ones we identify here, may be prokaryotic inventions. However, we cannot exclude the possibility that these bacterial lipid binding modules are mimicking currently unidentified membrane interacting domains within eukaryotic proteins. For example, *Vibrio parahaemolyticus* type III effector VopS contains a PX-type lipid-binding module that uses PIPs as folding substrate as the effector protein enters host cells^30^.

Intracellular pathogens must avoid the fusion of the degradative contents of lysosomes with their respective bacterial-containing vacuoles. Indeed, *Legionella* employs many of is nearly 350 effector proteins to subvert host endomembrane trafficking with several overlapping strategies. Owing to this redundancy, it has been difficult to identify cellular targets and functions of *Legionella* effector proteins. Here, we show that the membrane-targeting effector proteins Lpg0634^*Lp*^, Lpg1717^*Lp*^, Lpg1751^*Lp*^, and Ceg19^*Lp*^ appear to induce fragmentation of yeast vacuoles and to disrupt endolysosomal functions in mammalian cells. We speculate that these proteins may inhibit fusion of lysosomes with the LCV, yet further studies would be necessary to validate these results during infection. Thus, it will be important to determine how membrane targeting facilitates the enzymatic activity and function of individual effector proteins in eukaryotic cells and to further define the role of these host targeting mechanisms in the pathogenic system.

## Methods

### Molecular biology

The bacterial effector gene library was assembled from several sources and uses the entire open reading frames (ORFs) of effector genes (accession codes listed below) from the following sources: *Pseudomonas syringe* pv. *Tomato* (ATCC BAA-871D-5) and *Legionella pneumophila* Philadelphia-1 (ATCC 33152D-5), *Salmonella* Typhimurium LT2 (a gift from Jack Dixon, University of California San Diego), EHEC H7:O157 (a gift from Vanessa Sperandio, University of Texas Southwestern Medical Center), and *Shigella flexneri* M90T (a gift from Jack Dixon, University of California San Diego). *Bartonella henselae* Houston-1 effector proteins cloned into pENTR were a gift from Alexei Savchenko (University of Toronto). All pENTR effector gene clones were verified by DNA sequencing and are available upon request. Stop codons were introduced into bacterial effector proteins that displayed potential prenylation sites or PDZ-ligand sequences at the COOH-terminus (Supplementary Table 1) The Ras-rescue plasmid p3S0BL2 was a kind gift of Mark Lemmon (University of Pennsylvania)^13^. To facilitate the rapid transfer of bacterial effector genes into this plasmid, we inserted a Gateway expression cassette (Invitrogen) in between Ras and the HA tag. The resulting plasmid is named pRRD for plasmid Ras-Rescue DEST. The entire pENTR effector library was then recombined into the pRRD vector using LR Clonase II (Invitrogen) following manufacturer’s instructions. To monitor the subcellular localization of membrane-localized effectors in yeast, the p413Gal vector (gift from Dr. Ben Tu, University of Texas Southwestern Medical Center) was modified to contain the open reading fame of EGFP with a gateway expression cassette at its 3′ end. The 60 effectors positive in the Ras-Rescue screen were then gateway-cloned similarly as with the pRRD vector. For effectors purified with a strep-tag, effectors were first gateway-cloned into pCDNA3.1 GFP–strep destination vector. All constructs were verified by DNA sequencing.). All subcloning was performed by recombination using Gateway-adapted vectors following manufacturer’s instructions (Invitrogen) unless otherwise mentioned.

### Yeast-based assays

The following *S. cerevisiae* strains were used: INV*S*c1 (*MATa his3D1 leu2 trp1-289 ura3-52*) (Invitrogen), the yeast knockout strains ΔVPS34, ΔFAB1, and ΔLSB6 strains with genetic background BY4741 (MATa his3Δ1 leu2Δ0 met15Δ0 ura3Δ0) were kind gifts of Joel Goodman, University of Texas Southwestern Medical Center. The tetracycline-off strains MSS4, STT4, and PIK1 strains in the genetic background of R1158 (URA::CMV-tTA MATa his3-1 leu2-0 met15-0) were obtained through the Yeast Tet-Promoters Hughes Collection (Thermo scientific). The *cdc25ts* yeast strain 352-15A2 (*MATa*, *ade5*, *cdc25-2*, *his7*, *met10*, *trp1*, *ura3-52*) was a gift from Mark Lemmon, University of Pennsylvania, contains a temperature-sensitive allele of *CDC25* ^42^. BY4742 (MATα; his3Δ 1 leu2Δ 0 lys2Δ 0 ura3Δ 0) was a gift from Joel Goodman, University of Texas Southwestern Medical Center.

A standard lithium acetate protocol^43^ was used for transformation of all the strains except for CDC25ts, for which we used a modified LiAc protocol^13^. Briefly, a 5 mL overnight culture of *cdc25ts* yeast, grown at 25 °C, was diluted to 50 mL YPAD medium and shaken at 25 °C for 4 h. Cells were pelleted at 2000×*g* for 5 min in a room temperature centrifuge and then washed once with 50 mL TE. Cells were repelleted and then resuspended in 100 mM LiAc (2 mL). The LiAc: yeast suspension was incubated for 10 min at 25 °C. Next, 5 μL of carrier DNA (Clontech), 50 μL of LiAc:yeast suspension, and 350 μL of 40% PEG solution in 100 mM LiAc was added to each tube containing the miniprepped DNA. After mixing gently with a pipette, yeast were incubated for 30 min at 25 °C and then heat shocked at 42 °C for 15 min. Cells were pelleted, washed with TE (1 mL), plated on glucose plates lacking leucine, and allowed to grow for 4 days at 25 °C. To assay for rescue of the *cdc25ts* allele, transformants were resuspended in media lacking leucine and spotted in duplicate onto a plate grown at 25 °C and another grown at the selective temperature, 37 °C. Viability was scored between 3 and 10 days after growth as described previously^13^.

Yeast lysis for expression assays were performed as described previously^44^. Briefly, 2 mL overnight cultures were pelleted and resuspended in yeast lysis buffer (4% v/v NaOH, 0.5% v/v BME), vortexed for 1 min, and incubated on ice for 30 min. SDS page buffer was added in a 1:1 ratio and samples were boiled at 80 °C for 5 min. The insoluble fraction was pelleted and the soluble fraction was run on a 10% acrylamide gel and transferred onto nitrocellulose. The membrane was then blocked with 3% skim milk in TBS + 0.1% tween (TBST) for 1 h and probed with rabbit anti-Ras (Cell Signaling Technology, #3965) or mouse anti-HA (Cell Signaling Technology, #2367) at 1:1000 overnight at 4 °C. Appropriate HRP-conjugated secondary antibodies (Thermo Scientific #31430 and #31460) were incubated with the membrane for 30 min at room temperature. Membranes were developed with Supersignal Femto Chemiluminescent Substrate (Thermo Scientific #34095).

After 2 days, transformants were grown overnight in galactose media lacking histidine and visualized on a LSM 510 PASCAL scanning confocal microscope (Zeiss, Thornwood, NY, USA). For the tetracycline-off strains, transformants were grown overnight in glucose media lacking histidine with the appropriate amount of doxycycline (optimized with the yeast protein Osh2) for repression (50 μg/μL doxycycline for PIK1 and STT4 and 300 μg/μL doxycycline for MSS4). The next day, cultures were pelleted, washed with TE, and grown for another 24 h in galactose media lacking histidine with the same concentrations of doxycycline. On the third day, yeast were visualized on a LSM 510 PASCAL scanning confocal microscope (Zeiss).

For FM4-64 dye (Invitrogen) vacuole labeling, effectors of interest were expressed in BY4742 yeast strain overnight in galactose inducible media and 500 μL of the culture was pelleted at 2000 × *g* for 5 min and resuspended in YPAD. FM4-64 dye (8 µm) was incubated with the yeast for 20 min at 30 °C. Yeast were pelleted, washed once with YPAD, and incubated at 30 °C for 60 min. Cultures were subsequently washed with galactose inducible media, and resuspended in 50 μL of galactose inducible media for visualization.

### Mammalian cell studies

HeLa (ATCC CCL-2) and HEK293T cells (ATCC CRL-3216) were maintained in Dulbecco’s modified Eagle medium containing 10% (v/v) FBS, 2 mM glutamine, and 100 µg/mL penicillin/streptomycin (Thermo Scientific, Waltham, MA, USA) at 37 °C in a 5% CO_2_ incubator. HeLa cells were seeded onto glass coverslips overnight and transfected at 60–80% confluency with 1 μg of DNA using Fugene6 (Roche). After 16–18 h, cells were fixed in 3.7% formaldehyde and visualized on a LSM 510 PASCAL scanning confocal microscope (Zeiss). Colocalization studies were performed with a wide array of transfectable markers including the lipid binding domains of Spo20p (IDT gene synthesis), Osh2p (2 tandem copies of the PH domain; cDNA kindly provided by Dr. Scott Emr, Weill Cornell Medical Sciences), Akt (one PH domain; cDNA kindly provided by Dr. Michael White, University of Texas Southwestern Medical Center), were subcloned into pcDNA 3.1-GFP to generate constructs similar to what has been described previously^45–47^. pEGFP 2xPH^PLC^ plasmid was a gift from Dr. Kim Orth, University of Texas Southwestern Medical Center, and has been previously described^48, 49^. Cells were stained Alexa Fluor 350 Phalloidin (Fisher #A22281), Alexa Fluor 594 Phalloidin (Fisher #A12381), or antibody targeting GM130 (BD Biosciences #610822).

For live cell imaging experiments, HeLa cells were seeded onto glass bottom dishes (MatTek). Cells were transiently transfected with pcDNA 3.1 EGFP-IpgB1^E80A^ overnight and infected with mCherry-expressing *Shigella* M90T. Images were acquired every minute and images were corrected for photobleaching using ImageJ.

### Recombinant protein

For strep-tag purification of proteins, 10 cm dishes of HEK293T cells were transfected with 10 μg of plasmid DNA using calcium phosphate. After 24–48 h, cells were washed once with cold PBS and lysed in Mammalian Lysis Buffer (0.5% triton, 4 mM MgCl_2_ in 10 mM Tris pH 7.5). Strep-tagged effector proteins were purified through affinity chromatography by incubating streptactin beads (Qiagen) at 4 °C. Beads were washed three times before the protein was eluted in Strep-tag elution buffer (IBA). Western blots with HRP-conjugated anti-streptactin (IBA) confirmed expression.

### Lipid interactions

Bacterial effector proteins carrying a GFP–Strep-affinity tag were expressed in mammalian HEK293T cells and purified by streptactin agarose chromatography. PIP strips (Invitrogen) were blocked in 3% fatty acid free (faf) BSA in TBST for 1 h shaking at room temperature. The effector protein of interest was diluted in 750 μL of TBST + 3% fatty acid-free (faf) BSA and incubated with the PIP strip for 3 h. The membrane was washed four times with TBST + 3% faf-BSA and then incubated with HRP-conjugated anti-streptactin (IBA; 2-1502-001; 1:5000) for 45 min and washed before chemiluminescent detection. Protein–lipid interactions were detected by autoradiography or by western blot (HRP-Anti-streptactin; IBA).

### Accession codes for the effector proteins

*Pseudomonas syringe pv. tomato*. AvrPto1 NP_793764.1, HopA1 NP_795084.1, HopAA1-2 NP_794461.1, HopAB2 NP_792881.1, HopAG1 NP_790740.1, HopAH1 NP_790744.1, HopAH2-1 NP_793075.1, HopAI1 NP_790745.1, HopAM1-1 NP_790858, HopAO1 NP_794465.1, HopAQ1 NP_794448.1, HopAS1 NP_790323.1, HopC1 NP_790436.1, HopD YP_784364.1, HopE1 NP_794087, HopF2 NP_790351.1, HopG1 NP_794468, HopH1 NP_790435, HopI1 NP_794511, HopK1 NP_789904.1, HopM1 NP_791202.1, HopN1 NP_791197, HopO1-2 NP_794345, HopO1-3 NP_794343.1, HopP1 NP_792485, HopQ1-1 NP_790716, HopQ1-2 NP_794471.1, HopS1 NP_794348.1, HopT1-2 NP_794344.1, HopU1 NP_790350.1, HopV1 NP_794463.1, and HopY1 NP_789920.1.

*Salmonella* Typhimurium. AvrA NP_461786.1, PipB NP_460061.1, PipB2 NP_461706.1, SifA NP_460194.1, SifB NP_460561.1, SipB NP_461806.1, SipC NP_461805.1, SipD NP_461804.1, SopA NP_461011.1, SopB NP_460064.1, SopD NP_461866.1, SopD2 CBW17005, SpiC NP_460358.1, SptP NP_461799.1, SsaB NP_460358.1, SsaE NP_460361.1, SsaM NP_460378.1, SseE NP_460367.1, SseG NP_460370.1, SseI NP_461184.1, SseL NP_461229.2, SseJ NP_460590.1, SspH2 NP_461184.1, SteA NP_460542.1, SteB Q8ZPA6, and EHEC H7:O157.

EspB NP_290254.1, EspF NP_290250.1, EspG NP_290289.1, EspH NP_290264.1, EspJ NP_288436.1, EspK NP_287316.1, EspL1 NP_288154.1, EspL2 NP_289551.1, EspM1 NP_287949.1, EspM2 NP_289175.1, EspR1 NP_287686.1, EspR3 NP_288394.1, EspT NP_289175.1, EspW NP_289177.1, EspX1 NP_285716.1, EspX2 NP_286562.1, EspX4 NP_290672.1, EspX5 NP_290699.1, EspY1 NP_285753.1, EspY2 NP_285765.1, EspY3 NP_286160.1, Map NP_290262.1, NleA11 NP_287961.1, NleB1 NP_286532.1, NleC NP_286533.1, NleD NP_286535.1, NleF NP_287958.1, NleG7 NP_287535.1, NleH1 NP_286534.1, SepZ NP_290271.1, and TIR NP_286906.1.

*Legionella pneumophila*. Ceg7 YP_094281.1, Ceg9 YP_094300.1, Ceg10 YP_094338.1, Ceg18 YP_094932.1, Ceg19 YP_095154.1, Ceg23 YP_095648.1, Ceg29 YP_096417.1, Ceg33 YP_096596.1, CegC1 YP_094067.1 CegC3 YP_095177.1, CegC4 YP_096212.1, LegA2 YP_096227.1, LegA3 YP_096309.1, LegA5 YP_096331.1, LegA7 YP_094447.1, LegA8 YP_094731.1, LegA9 YP_094446.1, LegA10 YP_094093.1, LegA11 YP_094480.1, LegA12 YP_094527.1, LegA14 YP_096459.1, LegA15 YP_096463.1, LegAS4 YP_095745.1, LegAU13 YP_096157.1, LegC3 YP_095728.1, LegC4 YP_095969.1, LegC5 YP_095517.1, LegG1 YP_095992.1, LegG2 YP_094330.1, LegK2 YP_096150.1, LegK3 YP_096563.1, LegL1 YP_094979.1, LegL2 YP_095629.1, LegL3 YP_095687.1, LegL5 YP_095974.1, LegL7 YP_096408.1, LegLC4 YP_095964.1, LegLC8 YP_095907.1, LegP YP_096991.1, LegS2 YP_096188.1, LegU1 YP_094225.1, LegU2 YP_096825.1, LidA YP_094974.1, LirA YP_095976.1, LirB YP_095978.1, LirC YP_095979.1, LirD YP_095980.1, LirF YP_095982.1, lpg0045 YP_094100.1, lpg0081YP_094135.1, lpg0294 YP_094348.1, lpg0365 YP_094409.1, lpg0518 YP_094562.1, lpg0634 YP_094670.1, lpg0963 YP_094997.1, lpg1148 YP_095181.1, lpg1158 YP_095191.1, lpg1273 YP_095303.1, lpg1689 YP_095716.1, lpg1717 YP_095744.1, lpg1751 YP_095777.1, lpg2327 YP_096336.1, lpg2407 YP_096415.1, lpg2527 YP_096534.1, LpnE YP_096234.1, PieE YP_095985.1, PieF YP_095988.1, PpeB YP_095729.1, RalF YP_095966.1, SdjA YP_096515.1, SetA YP_095994.1, SidA YP_094657.1, SidB YP_095669.1, SidC YP_096518.1, SidD YP_096472.1, SidF YP_096589.1, SidG YP_095384.1, SidJ YP_096168.1, SidM YP_096471.1, VipA YP_094434.1, VipD YP_096826.1, VipF YP_094157.1, VpdA YP_096418.1, VpdB YP_095258.1, WipA YP_125080.1, WipB YP_094678.1, YlfA YP_096307.1, and YlfB YP_095901.1.

*Shigella flexneri*. IpaA AAK18443.1, IpaB AAK18446, IpaC AAK18445.1, IpaD AAK18444.1, IpaH1.4 AAK18594.1, IpaH 2.5 AAK18367, IpaH4.5 AAK18395, IpaH 7.8 AAK18394.1, IpaH 9.8 AAK18544, IpaJ AAK18440, IpgB1 CAC05805.1, IpgB2 CAC05777.1, IpgD AAK18452, OspB CAC05770.1, OspC1 CAC05790.1, OspC2 AAW64906, OspD1 AAW64782, OspD3 AAW64880, OspE1 AAW64916, OspE2 AAW64805, OspF AAW64770, OspG NP_085391, VirA AAK18501, BepA YP_034062.1, BepB YP_034064.1, BepC YP_034065.1, BepD YP_034066.1, BepE YP_034067.1, and BepF YP_034068.1.

### Data availability

The authors declare that all the relevant data supporting the findings of the study are available in this article and its Supplementary Information files, or from the corresponding author upon request.

## Electronic supplementary material

Supplementary Information
