# Supplementary material for: A systematic exploration of the interactions between bacterial effector proteins and host cell membranes
Source: Nat Commun. 2017 Sep 14;8:532. doi: 10.1038/s41467-017-00700-7 (PMC5599653; doi:10.1038/s41467-017-00700-7)
Supplement: Supplementary file 1 — Supplementary Information [file 41467_2017_700_MOESM1_ESM.pdf]

Description of Supplementary Files

File Name: Supplementary Information
Description: Supplementary Figures, Supplementary Tables and Supplementary References

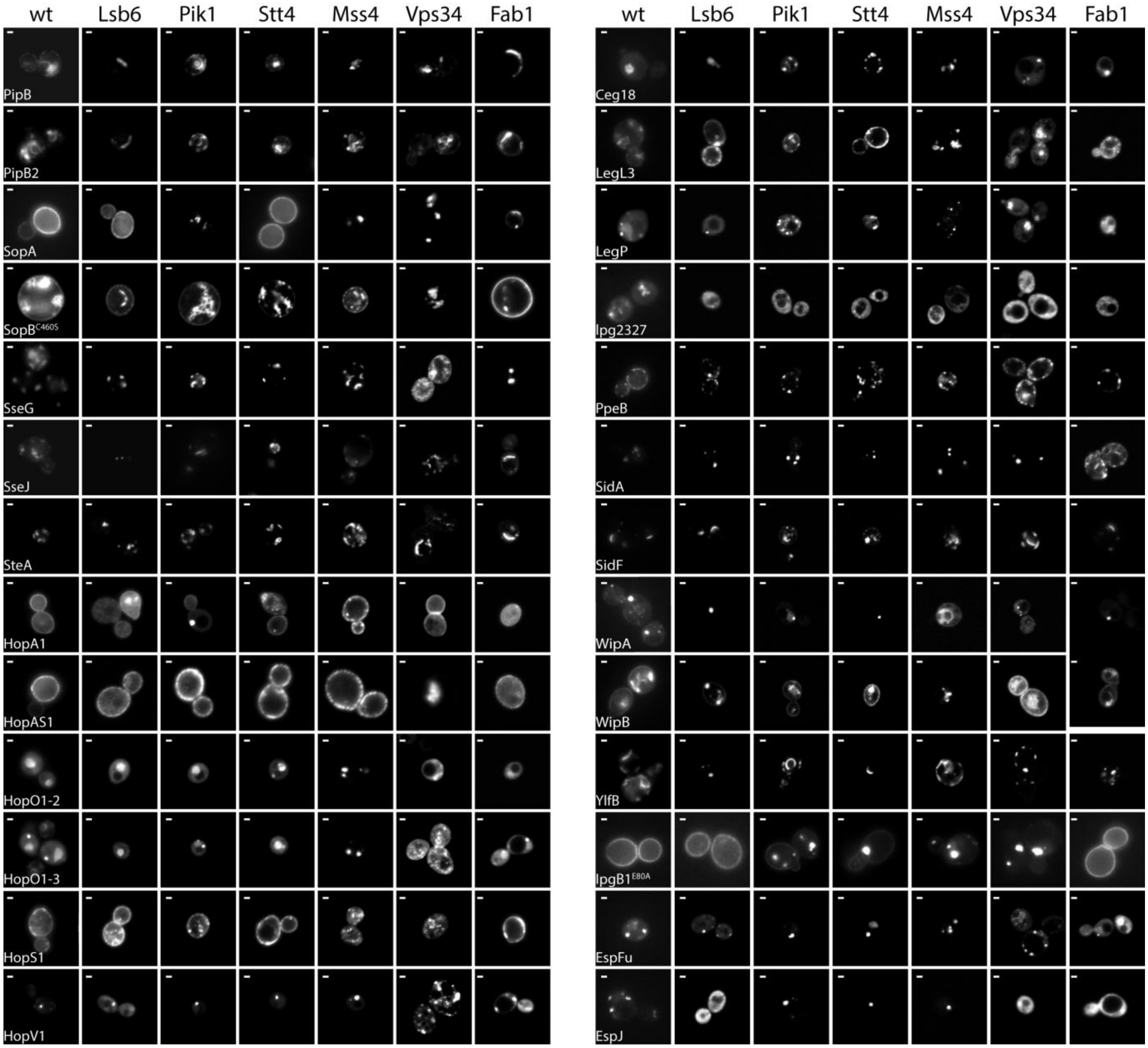

Supplementary Figure 1: PI kinase screen of GFP-tagged bacterial effector proteins.

Localization of GFP fused effector proteins were examined in the six PI kinase deletion or tet-off strains. Tet-off strains were repressed for 24 hours before the expression of each effector protein under a galactose inducible promoter. All images are representative of at least 75% of expressing cells for each of the 23 effectors that changed localization in one or more PI Kinase strains. Phosphoinositides do not appear to play a dominant role in membrane localization of the other effectors tested . Scale bar is 1 µm

A

Probe Legend

Spo20: Phosphatidic acid	PH-PLC: PI(4,5)P2
Lact-C2: Phosphatidylserine	PH-AKT: PI(3,4,5)P3
2xFYVE-EEA1: PI(3)P	GFP: Soluble
2XPH-Osh2p: PI(4)P	

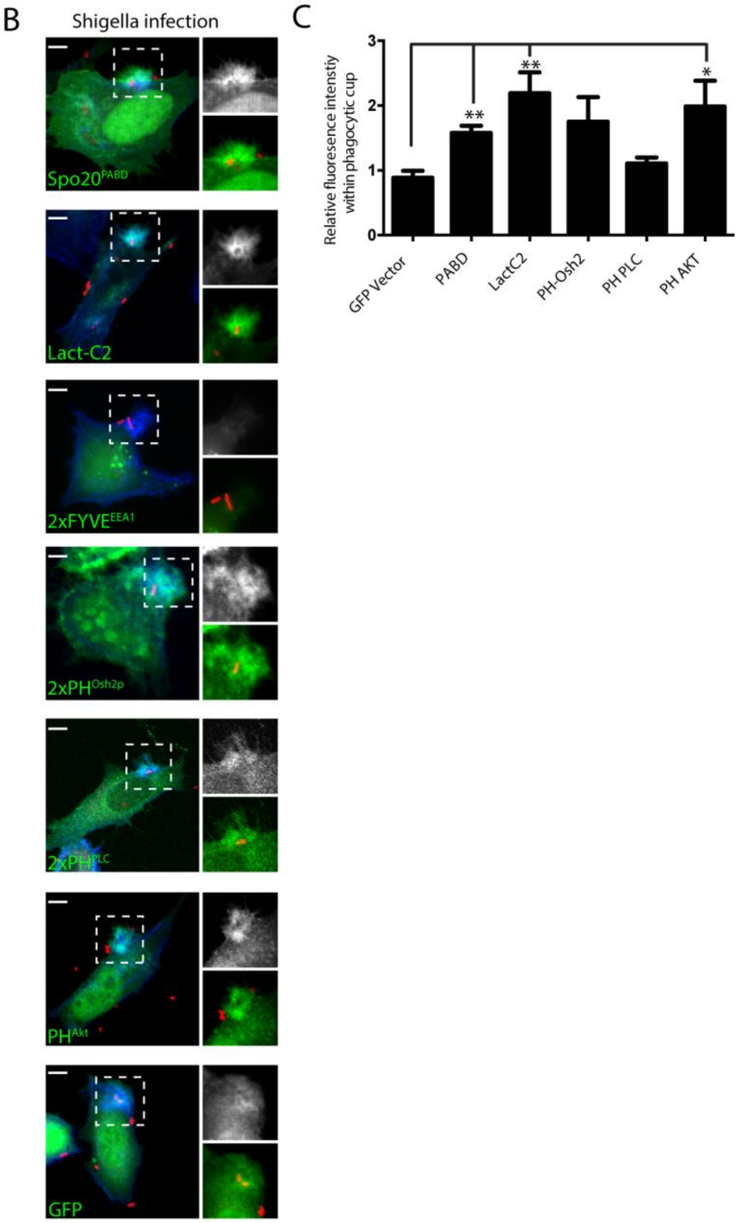

Supplementary Figure 2: Altered lipid composition at the plasma membrane during *Shigella* invasion

(a) Legend of lipid binding probes used.

(b) mCherry expressing *Shigella* M90T (red) infecting HeLa cells transiently transfected with indicated EGFP-tagged lipid binding probes (green) or GFP alone as a control. Phagocytic cup formation (F-actin in blue) was monitored similar to Figure 7. The box region is enlarged 2x showing the EGFP-tagged lipid probe (top) and the bacteria (red) and lipid probe (green) together. Scale bar is 5 µm. These data indicate that within the *Shigella* induced phagocytic cup, there are alterations in the plasma membrane phospholipid composition.

(c) Quantification of recruitment of lipid probes or GFP into the phagocytic cup during *Shigella* infection. Values reported as a fold increase of fluorescent signal in the phagocytic cup over the cytosol. 3 independent experiments and significance determined by a two-tailed t test, * $p < 0.05$, ** $p < 0.01$.

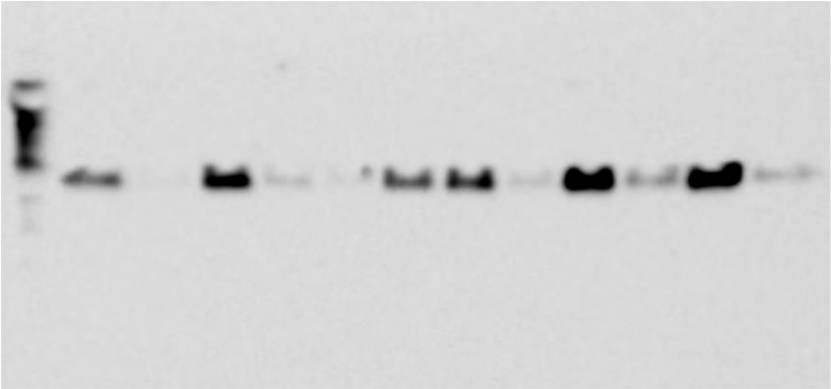

Supplementary Figure 3: Unaltered western blot used in manuscript

Supplementary Table 1: Bacterial effector proteins used in this study

Effector	Organism	Accession number	Expression in CDC25ts	Ras rescue	Literature
AvrPto1	*P. syringae*	NP_793764.1	yes	no	(1)
HopA1	*P. syringae*	NP_795084.1	yes	yes	(1)
HopAA1-2	*P. syringae*	NP_794461.1	yes	no	(1)
HopAB2	*P. syringae*	NP_792881.1	yes	no	(1)
HopAF1	*P. syringae*	NP_790740.1	yes	no	(1)
HopAG1	*P. syringae*	NP_790740.1	yes	no	(1)
HopAH1	*P. syringae*	NP_790744.1	yes	no	(1)
HopAH2-1	*P. syringae*	NP_793075.1	yes	no	(1)
HopAI1	*P. syringae*	NP_790745.1	yes	no	(1)
HopAM1-1	*P. syringae*	NP_790858	no	n/a	(1)
HopAO1	*P. syringae*	NP_794465.1	yes	no	(1)
HopAQ1	*P. syringae*	NP_794448.1	yes	no	(1)
HopAS-1	*P. syringae*	NP_790323.1	yes	yes	(2)
HopC1	*P. syringae*	NP_790436.1	yes	no	(1)
HopD	*P. syringae*	YP_784364.1	yes	no	(1)
HopE1	*P. syringae*	NP_794087	yes	no	(1)
HopF2	*P. syringae*	NP_790351.1	yes	no	(1)
HopG1	*P. syringae*	NP_794468	yes	no	(1)
HopH1	*P. syringae*	NP_790435	yes	no	(1)
HopI1	*P. syringae*	NP_794511	yes	no	(1)
HopK1	*P. syringae*	NP_789904.1	yes	no	(1)
HopM1	*P. syringae*	NP_791202.1	yes	no	(1)
HopN1	*P. syringae*	NP_791197	yes	no	(1)
HopO1-2	*P. syringae*	NP_794345	yes	yes	(1)
HopO1-3	*P. syringae*	NP_794343.1	yes	yes	(1)
HopP1	*P. syringae*	NP_792485	yes	no	(1)
HopQ1-1	*P. syringae*	NP_790716	yes	no	(1)
HopQ1-2	*P. syringae*	NP_794471.1	yes	no	(1)
HopS1	*P. syringae*	NP_794348.1	yes	yes	(1)
HopT1-2	*P. syringae*	NP_794344.1	yes	no	(1)
HopU1	*P. syringae*	NP_790350.1	yes	no	(1)
HopV1	*P. syringae*	NP_794463.1	yes	yes	(1)
HopY1	*P. syringae*	NP_789920.1	yes	no	(1)
AvrA	*S. typhimurium*	NP_461786.1	yes	no	(3)
PipB	*S. typhimurium*	NP_460061.1	yes	yes	(3)
PipB2	*S. typhimurium*	NP_461706.1	yes	yes	(3)
SifA*	*S. typhimurium*	NP_460194.1	yes	yes	(3)

SifB^{E199A}	*S. typhimurium*	NP_460561.1	yes	no	(4)
SipB	*S. typhimurium*	NP_461806.1	yes	no	(3)
SipC	*S. typhimurium*	NP_461805.1	yes	no	(3)
SipD	*S. typhimurium*	NP_461804.1	yes	no	(3)
SopA	*S. typhimurium*	NP_461011.1	yes	yes	(3)
SopB^{C460S}	*S. typhimurium*	NP_460064.1	yes	no	(3)
SopD	*S. typhimurium*	NP_461866.1	yes	no	(3)
SopD2	*S. typhimurium*	CBW17005	yes	no	(3)
SopE2^{G168V}	*S. typhimurium*	NP_460811.1	yes	yes	(3)
SpiC	*S. typhimurium*	NP_460358.1	yes	no	(3)
SptP	*S. typhimurium*	NP_461799.1	yes	no	(3)
SseE	*S. typhimurium*	NP_460367.1	yes	no	(5)
SseF	*S. typhimurium*	NP_460369.1	no	n/a	(3)
SseG	*S. typhimurium*	NP_460370.1	yes	yes	(3)
SseI	*S. typhimurium*	NP_461184.1	yes	no	(6)
SseL	*S. typhimurium*	NP_461229.2	yes	no	(3)
SseJ	*S. typhimurium*	NP_460590.1	yes	yes	(3)
SspH2	*S. typhimurium*	NP_461184.1	yes	no	(3)
SteA	*S. typhimurium*	NP_460542.1	yes	yes	(7)
SteB	*S. typhimurium*	Q8ZPA6	no	n/a	(3)
EspB	*EHEC H7:O157*	NP_290254.1	yes	no	(8)
EspF	*EHEC H7:O157*	NP_290250.1	yes	yes	(8)
EspFu	*EHEC H7:O157*	NP_286906.1	yes	yes	(8)
EspG	*EHEC H7:O157*	NP_290289.1	yes	no	(8)
EspH*	*EHEC H7:O157*	NP_290264.1	yes	yes	(8)
EspJ	*EHEC H7:O157*	NP_288436.1	yes	yes	(8)
EspK	*EHEC H7:O157*	NP_287316.1	yes	no	(8)

EspL1	*EHEC H7:O157*	NP_288154.1	yes	no	(8)
EspL2	*EHEC H7:O157*	NP_289551.1	no	n/a	(8)
EspM2^{E70A}	*EHEC H7:O157*	NP_289175.1	yes	no	(8)
EspR1	*EHEC H7:O157*	NP_287686.1	yes	no	(8)
EspR3	*EHEC H7:O157*	NP_288394.1	yes	no	(8)
EspT^{E67A}	*EHEC H7:O158*	NP_289175.1	yes	no	(9)
EspW	*EHEC H7:O157*	NP_289177.1	yes	no	(8)
EspX1	*EHEC H7:O157*	NP_285716.1	no	n/a	(8)
EspX2	*EHEC H7:O157*	NP_286562.1	yes	no	(8)
EspX4	*EHEC H7:O157*	NP_290672.1	no	n/a	(8)
EspX5	*EHEC H7:O157*	NP_290699.1	no	n/a	(8)
EspX7	*EHEC H7:O157*	NP_290699.1	yes	no	(8)
EspY1	*EHEC H7:O157*	NP_285753.1	yes	no	(8)
EspY2	*EHEC H7:O157*	NP_285765.1	yes	no	(8)
EspY3	*EHEC H7:O157*	NP_286160.1	yes	no	(8)
Map	*EHEC H7:O157*	NP_290262.1	yes	no	(8)
NleA11	*EHEC H7:O157*	NP_287961.1	yes	no	(8)
NleB1*	*EHEC H7:O157*	NP_286532.1	yes	no	(8)
NleC	*EHEC H7:O157*	NP_286533.1	yes	no	(8)
NleD	*EHEC H7:O157*	NP_286535.1	yes	no	(8)
NleF	*EHEC H7:O157*	NP_287958.1	yes	no	(8)
NleG7	*EHEC H7:O157*	NP_287535.1	yes	no	(8)
NleH1	*EHEC H7:O157*	NP_286534.1	yes	yes	(8)
SepZ	*EHEC H7:O157*	NP_290271.1	no	n/a	(8)
Tir	*EHEC H7:O157*	NP_286906.1	yes	yes	(8)
Ceg7	*L. pneumophila*	YP_094281.1	yes	yes	(10)
Ceg9	*L. pneumophila*	YP_094300.1	yes	yes	(11)

Ceg10	*L. pneumophila*	YP_094338.1	yes	no	(12)
Ceg18	*L. pneumophila*	YP_094932.1	yes	yes	(13)
Ceg19	*L. pneumophila*	YP_095154.1	yes	yes	(11)
Ceg23	*L. pneumophila*	YP_095648.1	yes	no	(12)
Ceg29	*L. pneumophila*	YP_096417.1	yes	no	(12)
Ceg33	*L. pneumophila*	YP_096596.1	yes	no	(12)
CegC1	*L. pneumophila*	YP_094067.1	yes	yes	(14)
CegC3	*L. pneumophila*	YP_095177.1	yes	no	(12)
CegC4	*L. pneumophila*	YP_096212.1	yes	yes	(14)
LegA3	*L. pneumophila*	YP_096309.1	yes	no	(15)
LegA5	*L. pneumophila*	YP_096331.1	yes	yes	(15)
LegA7	*L. pneumophila*	YP_094447.1	yes	no	(13)
LegA8	*L. pneumophila*	YP_094731.1	yes	no	(11)
LegA9	*L. pneumophila*	YP_094446.1	yes	no	(13)
LegA10	*L. pneumophila*	YP_094093.1	yes	no	(15)
LegA11	*L. pneumophila*	YP_094480.1	yes	no	(15)
LegA12	*L. pneumophila*	YP_094527.1	yes	yes	(15)
LegA14	*L. pneumophila*	YP_096459.1	yes	no	(15)
LegA15	*L. pneumophila*	YP_096463.1	yes	no	(15)
LegAS4	*L. pneumophila*	YP_095745.1	yes	no	(15)
LegAU13	*L. pneumophila*	YP_096157.1	yes	no	(11)
LegC3	*L. pneumophila*	YP_095728.1	yes	yes	(11)
LegC4	*L. pneumophila*	YP_095969.1	yes	yes	(15)
LegG1	*L. pneumophila*	YP_095992.1	yes	no	(15)
LegG2	*L. pneumophila*	YP_094330.1	yes	no	(15)
LegK2	*L. pneumophila*	YP_096150.1	yes	no	(15)
LegK3	*L. pneumophila*	YP_096563.1	yes	no	(15)

LegL1	*L. pneumophila*	YP_094979.1	yes	yes	(15)
LegL2	*L. pneumophila*	YP_095629.1	no	no	(15)
LegL3	*L. pneumophila*	YP_095687.1	yes	yes	(15)
LegL5	*L. pneumophila*	YP_095974.1	yes	no	(15)
LegL7	*L. pneumophila*	YP_096408.1	yes	no	(15)
LegLC4	*L. pneumophila*	YP_095964.1	yes	no	(15)
LegLC8	*L. pneumophila*	YP_095907.1	yes	yes	(15)
LegP	*L. pneumophila*	YP_096991.1	yes	yes	(15)
LegS2	*L. pneumophila*	YP_096188.1	yes	yes	(15)
LegU1	*L. pneumophila*	YP_094225.1	yes	no	(15)
LegU2	*L. pneumophila*	YP_096825.1	yes	no	(11)
LidA	*L. pneumophila*	YP_094974.1	yes	no	(11)
LirA	*L. pneumophila*	YP_095976.1	yes	yes	(12)
LirB	*L. pneumophila*	YP_095978.1	yes	no	(12)
LirC	*L. pneumophila*	YP_095979.1	no	n/a	(12)
LirD	*L. pneumophila*	YP_095980.1	no	n/a	(12)
LirF	*L. pneumophila*	YP_095982.1	yes	no	(12)
lpg0045	*L. pneumophila*	YP_094100.1	yes	no	(16)
lpg0081	*L. pneumophila*	YP_094135.1	yes	no	(16)
lpg0294	*L. pneumophila*	YP_094348.1	yes	no	(16)
lpg0365	*L. pneumophila*	YP_094409.1	yes	no	(14)
lpg0518	*L. pneumophila*	YP_094562.1	yes	yes	(14)
lpg0634	*L. pneumophila*	YP_094670.1	yes	yes	(14)
lpg0963	*L. pneumophila*	YP_094997.1	yes	no	(14)
lpg1148	*L. pneumophila*	YP_095181.1	yes	yes	(12)
lpg1158	*L. pneumophila*	YP_095191.1	yes	yes	(13)
lpg1273	*L. pneumophila*	YP_095303.1	yes	yes	(16)

lpg1689	*L. pneumophila*	YP_095716.1	yes	no	(14)
lpg1717	*L. pneumophila*	YP_095744.1	yes	yes	(17)
lpg1751	*L. pneumophila*	YP_095777.1	yes	yes	(16)
lpg2327	*L. pneumophila*	YP_096336.1	yes	yes	(13)
lpg2407	*L. pneumophila*	YP_096415.1	yes	yes	(12)
lpg2527	*L. pneumophila*	YP_096534.1	yes	no	(14)
LpnE	*L. pneumophila*	YP_096234.1	no	n/a	(11)
PieE	*L. pneumophila*	YP_095985.1	yes	yes	(18)
PieF	*L. pneumophila*	YP_095988.1	yes	no	(18)
PpeB	*L. pneumophila*	YP_095729.1	yes	yes	(16)
RalF	*L. pneumophila*	YP_095966.1	yes	no	(11)
SdjA	*L. pneumophila*	YP_096515.1	yes	no	(18)
SetA	*L. pneumophila*	YP_095994.1	yes	no	(11)
SidA	*L. pneumophila*	YP_094657.1	yes	yes	(13)
SidB	*L. pneumophila*	YP_095669.1	yes	yes	(19)
SidD	*L. pneumophila*	YP_096472.1	yes	no	(12)
SidF	*L. pneumophila*	YP_096589.1	yes	yes	(11)
SidG	*L. pneumophila*	YP_095384.1	yes	no	(15)
SidJ	*L. pneumophila*	YP_096168.1	yes	yes	(11)
SidM	*L. pneumophila*	YP_096471.1	no	n/a	(11)
VipA	*L. pneumophila*	YP_094434.1	yes	yes	(11)
VipD	*L. pneumophila*	YP_096826.1	yes	no	(11)
VipF	*L. pneumophila*	YP_094157.1	yes	no	(20)
VpdB	*L. pneumophila*	YP_095258.1	yes	no	(18)
WipA	*L. pneumophila*	YP_125080.1	yes	yes	(18)
WipB	*L. pneumophila*	YP_094678.1	yes	yes	(18)
YifA	*L. pneumophila*	YP_096307.1	yes	yes	(11)

YifB	*L. pneumophila*	YP_095901.1	yes	yes	(11)
IpaA	*S. flexneri*	AAK18443.1	yes	no	(21)
IpaB	*S. flexneri*	AAK18446	yes	no	(21)
IpaH1.4	*S. flexneri*	AAK18594.1	yes	no	(21)
IpaH2.5	*S. flexneri*	AAK18367	yes	no	(21)
IpaH4.5	*S. flexneri*	AAK18395	yes	no	(21)
IpaH7.8	*S. flexneri*	AAK18394.1	yes	no	(21)
IpaH9.8	*S. flexneri*	AAK18544	yes	no	(21)
IpaJ	*S. flexneri*	AAK18440	no	n/a	(21)
IpgB1^{E80A}	*S. flexneri*	CAC05805.1	yes	yes	(21)
IpgB2^{E64A}	*S. flexneri*	CAC05777.1	yes	no	(21)
IpgD^{C439S}	*S. flexneri*	AAK18452	yes	yes	(21)
OspB	*S. flexneri*	CAC05770.1	yes	no	(21)
OspC1	*S. flexneri*	CAC05790.1	yes	no	(21)
OspC2	*S. flexneri*	AAW64906	yes	no	(21)
OspD1	*S. flexneri*	AAW64782	yes	no	(21)
OspD3	*S. flexneri*	AAW64880	yes	no	(21)
OspE1	*S. flexneri*	AAW64916	yes	no	(21)
OspE2	*S. flexneri*	AAW64805	yes	no	(21)
OspF	*S. flexneri*	AAW64770	yes	no	(21)
OspG	*S. flexneri*	NP_085391	yes	no	(21)
VirA	*S. flexneri*	AAK18501	yes	no	(21)
BepA	*B. henselae*	YP_034062.1	yes	no	(22)
BepB	*B. henselae*	YP_034064.1	yes	no	(22)
BepC	*B. henselae*	YP_034065.1	yes	no	(22)
BepD	*B. henselae*	YP_034066.1	yes	yes	(22)
BepE	*B. henselae*	YP_034067.1	yes	yes	(22)
BepF	*B. henselae*	YP_034068.1	yes	no	(22)

Supplemental Table 2: Membrane localization, enzymatic activity, and host substrate of the 60 membrane localized effectors.

Effector	Organism	Yeast Localization	Mammalian Localization	Mechanism of membrane localization	Enzymatic Activity/Function	Substrate/Eukaryotic Interactors
PipB	*S. typhimurium*	Endoplasmic reticulum	endoplasmic reticulum (23) and phagosome (24)			
PipB2	*S. typhimurium*	Vacuole	endoplasmic reticulum (23)			kinesin light chain (25)
SifA	*S. typhimurium*	Plasma membrane	lysosomes (26)	prenylation (27, 28)		RhoA (29) and SKIP (27)
SopA	*S. typhimurium*	Plasma membrane	mitochondria (30)		E3 Ubiquitin ligase (31)	Trim56 and Trim65 (32)
SopB	*S. typhimurium*	Endoplasmic reticulum	early endosomes (33)		phosphatidylinositol phosphatase (34, 35) and guanine nucleotide dissociation inhibitor (36)	Cdc42 (36)
SopE2	*S. typhimurium*	Plasma membrane	Salmonella containing vacuole (37)		guanine nucleotide exchange factor (38)	Cdc42 (38)
SseG	*S. typhimurium*	Unidentified	Salmonella containing vacuole and Golgi apparatus (39)	transmembrane domain (39)		ACBD3 (40)
SseJ	*S. typhimurium*	Unidentified	Salmonella containing vacuole and late endosomes/lysosomes (41)	interactions with RhoA (42)	glycerophospholipid-cholesterol acyl transferase (43)	RhoA (29)
SteA	*S. typhimurium*	Unidentified	Golgi apparatus (7) and Salmonella containing vacuole and SIFs (44)	PI(4)P binding (44)		

EspF	*EHEC H7:0157*	Unidentified	mitochondria (45) and clathrin coated pits (46)			Abcf2 (47), N-WASP (46), and Snx9 (46, 48)
EspFu	*EHEC H7:0158*	Unidentified	unknown			N-WASP (49) and IRSp53 (50, 51)
EspH	*EHEC H7:0159*	Unidentified	plasma membrane (52)			Rho family GEFs (53)
EspJ	*EHEC H7:0160*	Unidentified	mitochondria (54)		ADP ribosyltransferase (55)	Src (55)
NleH	*EHEC H7:0161*	Plasma membrane	plasma membrane (56)		Kinase (57)	CRKL (58)
Tir	*EHEC H7:0162*	Unidentified	plasma membrane (59)	transmembrane (60)	receptor (59)	IRSp53 (50, 51)
Ceg7	*L. peumophila*	Unidentified	unknown			
Ceg9	*L. peumophila*	Unidentified	endoplasmic reticulum (10)			Rtn4 (61)
Ceg18	*L. peumophila*	Unidentified	unknown			
Ceg19	*L. peumophila*	Vacuole	late endosomes/lysosomes (10)			
CegC1	*L. peumophila*	Unidentified	unknown			phospholipase (62)
CegC4	*L. peumophila*	Cytosol	unknown			
LegA5	*L. peumophila*	Unidentified	unknown			
LegA12	*L. peumophila*	Unidentified	unknown			
LegC3	*L. peumophila*	Unidentified	endoplasmic reticulum (63)	transmembrane (63)	Q-SNARE mimics (64)	VAMP4 (64)

LegL1	*L. peumophila*	Cytosol	unknown			
LegL3	*L. peumophila*	Unidentified	unknown			
LegLC8	*L. peumophila*	Cytosol	unknown			
LegP	*L. peumophila*	Unidentified	unknown			
LirA	*L. peumophila*	Unidentified	unknown			
lpg0518	*L. peumophila*	Unidentified	unknown			
lpg0634	*L. peumophila*	Vacuole	unknown			
lpg1148	*L. peumophila*	Unidentified	unknown			
lpg1158	*L. peumophila*	Unidentified	unknown			
lpg1273	*L. peumophila*	Unidentified	unknown			
lpg1717	*L. peumophila*	Vacuole	unknown			
lpg1751	*L. peumophila*	Vacuole	unknown			
lpg2327	*L. peumophila*	Unidentified	Unknown			
lpg2407	*L. peumophila*	Unidentified	Unknown			
PieE	*L. peumophila*	Golgi	endoplasmic reticulum and Legionella containing vacuole (65)	transmembrane (65)		Rab GTPases (65)
PpeB	*L. peumophila*	Unidentified	unknown			

SidA	*L. peumophila*	Unidentified	unknown			
SidB	*L. peumophila*	Unidentified	unknown			
SidF	*L. peumophila*	Unidentified	Legionella containing vacuole (66)	transmembrane (66)	PIP phosphatase (66)	BNIP3 (67) and Bcl-rambo (67)
SidJ	*L. peumophila*	Golgi	unknown			
VipA	*L. peumophila*	Vacuole	early endosomes (68)		actin nucleator (68)	actin (68)
WipA	*L. peumophila*	Unidentified	unknown			
WipB	*L. peumophila*	Endoplasmic reticulum	unknown			
YlfA/LegC7	*L. peumophila*	Vacuole	endoplasmic reticulum (63)	transmembrane (63)	Q-SNARE mimics (64)	VAMP4 (64)
YlfB/LegC2	*L. peumophila*	Unidentified	endoplasmic reticulum (63)	transmembrane (63)	Q-SNARE mimics (64)	VAMP4 (64)
LegS2	*L. peumophila*	Unidentified	mitochondria (69)		sphingosine 1-phosphate lyase (69)	
BepD	*B. henselae*	Plasma membrane	plasma membrane (70)			
BepE	*B. henselae*	Plasma membrane	puncta (22)			
IpgB1	*S. flexneri*	Plasma membrane	plasma membrane (71)		guanine nucleotide exchange factor (13)	Rac1 (13)and ELMO (72)
IpgD	*S. flexneri*	Plasma membrane	plasma membrane (73)		phosphatidylinositol phosphatase (73)	
HopA1	*P. syringae*	Plasma membrane	unknown			
HopAS1	*P. syringae*	Plasma membrane	unknown			

HopO1-2	*P. syringae*	Nucleus	unknown			
HopO1-3	*P. syringae*	Unidentified	unknown			
HopS1	*P. syringae*	Plasma membrane	unknown			
HopV1	*P. syringae*	Unidentified	unknown			

Supplementary References:

1. Cunnac S, Lindeberg M, Collmer A. Pseudomonas syringae type III secretion system effectors: repertoires in search of functions. Curr Opin Microbiol. 2009;12(1):53-60. Epub 2009/01/23. doi: 10.1016/j.mib.2008.12.003. PubMed PMID: 19168384.
2. Sohn KH, Saucet SB, Clarke CR, Vinatzer BA, O'Brien HE, Guttman DS, et al. HopAS1 recognition significantly contributes to Arabidopsis nonhost resistance to Pseudomonas syringae pathogens. New Phytol. 2012;193(1):58-66. Epub 2011/11/04. doi: 10.1111/j.1469-8137.2011.03950.x. PubMed PMID: 22053875.
3. Agbor TA, McCormick BA. Salmonella effectors: important players modulating host cell function during infection. Cell Microbiol. 2011;13(12):1858-69. Epub 2011/10/10. doi: 10.1111/j.1462-5822.2011.01701.x. PubMed PMID: 21902796; PubMed Central PMCID: PMCPMC3381885.
4. Papezova K, Gregorova D, Jonuschies J, Rychlik I. Ordered expression of virulence genes in Salmonella enterica serovar typhimurium. Folia Microbiol (Praha). 2007;52(2):107-14. doi: 10.1007/BF02932148. PubMed PMID: 17575908.
5. Coombes BK, Brown NF, Valdez Y, Brumell JH, Finlay BB. Expression and secretion of Salmonella pathogenicity island-2 virulence genes in response to acidification exhibit differential requirements of a functional type III secretion apparatus and SsaL. J Biol Chem. 2004;279(48):49804-15. Epub 2004/09/20. doi: 10.1074/jbc.M404299200. PubMed PMID: 15383528.
6. Ehrbar K, Hardt WD. Bacteriophage-encoded type III effectors in Salmonella enterica subspecies 1 serovar Typhimurium. Infect Genet Evol. 2005;5(1):1-9. doi: 10.1016/j.meegid.2004.07.004. PubMed PMID: 15567133.
7. Geddes K, Worley M, Niemann G, Heffron F. Identification of new secreted effectors in Salmonella enterica serovar Typhimurium. Infect Immun. 2005;73(10):6260-71. doi: 10.1128/IAI.73.10.6260-6271.2005. PubMed PMID: 16177297; PubMed Central PMCID: PMCPMC1230965.
8. Tobe T, Beatson SA, Taniguchi H, Abe H, Bailey CM, Fivian A, et al. An extensive repertoire of type III secretion effectors in Escherichia coli O157 and the role of lambdoid phages in their dissemination. Proc Natl Acad Sci U S A. 2006;103(40):14941-6. Epub 2006/09/21. doi: 10.1073/pnas.0604891103. PubMed PMID: 16990433; PubMed Central PMCID: PMCPMC1595455.
9. Bulgin R, Arbeloa A, Goulding D, Dougan G, Crepin VF, Raymond B, et al. The T3SS effector EspT defines a new category of invasive enteropathogenic E. coli (EPEC) which form intracellular actin pedestals. PLoS Pathog. 2009;5(12):e1000683. Epub 2009/12/11. doi: 10.1371/journal.ppat.1000683. PubMed PMID: 20011125; PubMed Central PMCID: PMCPMC2782363.
10. Heidtman M, Chen EJ, Moy MY, Isberg RR. Large-scale identification of Legionella pneumophila Dot/Icm substrates that modulate host cell vesicle trafficking pathways. Cell Microbiol. 2009;11(2):230-48. Epub 2008/10/30. doi: 10.1111/j.1462-5822.2008.01249.x. PubMed PMID: 19016775; PubMed Central PMCID: PMCPMC2744955.
11. Hubber A, Roy CR. Modulation of host cell function by Legionella pneumophila type IV effectors. Annu Rev Cell Dev Biol. 2010;26:261-83. doi: 10.1146/annurev-cellbio-100109-104034. PubMed PMID: 20929312.
12. Zusman T, Degtyar E, Segal G. Identification of a hypervariable region containing new Legionella pneumophila Icm/Dot translocated substrates by using the conserved icmQ regulatory signature. Infect Immun. 2008;76(10):4581-91. Epub 2008/08/11. doi: 10.1128/IAI.00337-08. PubMed PMID: 18694969; PubMed Central PMCID: PMCPMC2546816.

13. Huang L, Boyd D, Amyot WM, Hempstead AD, Luo ZQ, O'Connor TJ, et al. The E Block motif is associated with Legionella pneumophila translocated substrates. Cell Microbiol. 2011;13(2):227-45. Epub 2010/11/03. doi: 10.1111/j.1462-5822.2010.01531.x. PubMed PMID: 20880356; PubMed Central PMCID: PMCPMC3096851.
14. Lifshitz Z, Burstein D, Peeri M, Zusman T, Schwartz K, Shuman HA, et al. Computational modeling and experimental validation of the Legionella and Coxiella virulence-related type-IVB secretion signal. Proc Natl Acad Sci U S A. 2013;110(8):E707-15. Epub 2013/02/04. doi: 10.1073/pnas.1215278110. PubMed PMID: 23382224; PubMed Central PMCID: PMCPMC3581968.
15. de Felipe KS, Pampou S, Jovanovic OS, Pericone CD, Ye SF, Kalachikov S, et al. Evidence for acquisition of Legionella type IV secretion substrates via interdomain horizontal gene transfer. J Bacteriol. 2005;187(22):7716-26. doi: 10.1128/JB.187.22.7716-7726.2005. PubMed PMID: 16267296; PubMed Central PMCID: PMCPMC1280299.
16. Burstein D, Amaro F, Zusman T, Lifshitz Z, Cohen O, Gilbert JA, et al. Genomic analysis of 38 Legionella species identifies large and diverse effector repertoires. Nat Genet. 2016;48(2):167-75. Epub 2016/01/11. doi: 10.1038/ng.3481. PubMed PMID: 26752266; PubMed Central PMCID: PMCPMC5050043.
17. Franco IS, Shuman HA, Charpentier X. The perplexing functions and surprising origins of Legionella pneumophila type IV secretion effectors. Cell Microbiol. 2009;11(10):1435-43. Epub 2009/06/26. doi: 10.1111/j.1462-5822.2009.01351.x. PubMed PMID: 19563462.
18. Ninio S, Roy CR. Effector proteins translocated by Legionella pneumophila: strength in numbers. Trends Microbiol. 2007;15(8):372-80. Epub 2007/07/13. doi: 10.1016/j.tim.2007.06.006. PubMed PMID: 17632005.
19. Brüggemann H, Hagman A, Jules M, Sismeiro O, Dillies MA, Gouyette C, et al. Virulence strategies for infecting phagocytes deduced from the in vivo transcriptional program of Legionella pneumophila. Cell Microbiol. 2006;8(8):1228-40. doi: 10.1111/j.1462-5822.2006.00703.x. PubMed PMID: 16882028.
20. Shohdy N, Efe JA, Emr SD, Shuman HA. Pathogen effector protein screening in yeast identifies Legionella factors that interfere with membrane trafficking. Proc Natl Acad Sci U S A. 2005;102(13):4866-71. Epub 2005/03/21. doi: 10.1073/pnas.0501315102. PubMed PMID: 15781869; PubMed Central PMCID: PMCPMC555709.
21. Parsot C. Shigella type III secretion effectors: how, where, when, for what purposes? Curr Opin Microbiol. 2009;12(1):110-6. Epub 2009/01/20. doi: 10.1016/j.mib.2008.12.002. PubMed PMID: 19157960.
22. Schulein R, Guye P, Rhomberg TA, Schmid MC, Schröder G, Vergunst AC, et al. A bipartite signal mediates the transfer of type IV secretion substrates of Bartonella henselae into human cells. Proc Natl Acad Sci U S A. 2005;102(3):856-61. Epub 2005/01/10. doi: 10.1073/pnas.0406796102. PubMed PMID: 15642951; PubMed Central PMCID: PMCPMC545523.
23. Knodler LA, Vallance BA, Hensel M, Jäckel D, Finlay BB, Steele-Mortimer O. Salmonella type III effectors PipB and PipB2 are targeted to detergent-resistant microdomains on internal host cell membranes. Mol Microbiol. 2003;49(3):685-704. PubMed PMID: 12864852.
24. Núñez-Hernández C, Alonso A, Pucciarelli MG, Casadesús J, García-del Portillo F. Dormant intracellular Salmonella enterica serovar Typhimurium discriminates among Salmonella pathogenicity island 2 effectors to persist inside fibroblasts. Infect Immun. 2014;82(1):221-32. Epub 2013/10/21. doi: 10.1128/IAI.01304-13. PubMed PMID: 24144726; PubMed Central PMCID: PMCPMC3911833.
25. Henry T, Couillault C, Rockenfeller P, Boucrot E, Dumont A, Schroeder N, et al. The Salmonella effector protein PipB2 is a linker for kinesin-1. Proc Natl Acad Sci U S A. 2006;103(36):13497-502. Epub

2006/08/25. doi: 10.1073/pnas.0605443103. PubMed PMID: 16938850; PubMed Central PMCID: PMCPMC1569191.
26. Stein MA, Leung KY, Zwick M, Garcia-del Portillo F, Finlay BB. Identification of a Salmonella virulence gene required for formation of filamentous structures containing lysosomal membrane glycoproteins within epithelial cells. Mol Microbiol. 1996;20(1):151-64. PubMed PMID: 8861213.
27. Boucrot E, Beuzón CR, Holden DW, Gorvel JP, Méresse S. Salmonella typhimurium SifA effector protein requires its membrane-anchoring C-terminal hexapeptide for its biological function. J Biol Chem. 2003;278(16):14196-202. Epub 2003/02/05. doi: 10.1074/jbc.M207901200. PubMed PMID: 12574170.
28. Reinicke AT, Hutchinson JL, Magee AI, Mastroeni P, Trowsdale J, Kelly AP. A Salmonella typhimurium effector protein SifA is modified by host cell prenylation and S-acylation machinery. J Biol Chem. 2005;280(15):14620-7. Epub 2005/02/14. doi: 10.1074/jbc.M500076200. PubMed PMID: 15710609.
29. Ohlson MB, Huang Z, Alto NM, Blanc MP, Dixon JE, Chai J, et al. Structure and function of Salmonella SifA indicate that its interactions with SKIP, SseJ, and RhoA family GTPases induce endosomal tubulation. Cell Host Microbe. 2008;4(5):434-46. doi: 10.1016/j.chom.2008.08.012. PubMed PMID: 18996344; PubMed Central PMCID: PMCPMC2658612.
30. Layton AN, Brown PJ, Galyov EE. The Salmonella translocated effector SopA is targeted to the mitochondria of infected cells. J Bacteriol. 2005;187(10):3565-71. doi: 10.1128/JB.187.10.3565-3571.2005. PubMed PMID: 15866946; PubMed Central PMCID: PMCPMC1112013.
31. Zhang Y, Higashide WM, McCormick BA, Chen J, Zhou D. The inflammation-associated Salmonella SopA is a HECT-like E3 ubiquitin ligase. Mol Microbiol. 2006;62(3):786-93. doi: 10.1111/j.1365-2958.2006.05407.x. PubMed PMID: 17076670.
32. Kamanova J, Sun H, Lara-Tejero M, Galán JE. The Salmonella Effector Protein SopA Modulates Innate Immune Responses by Targeting TRIM E3 Ligase Family Members. PLoS Pathog. 2016;12(4):e1005552. Epub 2016/04/08. doi: 10.1371/journal.ppat.1005552. PubMed PMID: 27058235; PubMed Central PMCID: PMCPMC4825927.
33. Dukes JD, Lee H, Hagen R, Reaves BJ, Layton AN, Galyov EE, et al. The secreted Salmonella dublin phosphoinositide phosphatase, SopB, localizes to PtdIns(3)P-containing endosomes and perturbs normal endosome to lysosome trafficking. Biochem J. 2006;395(2):239-47. doi: 10.1042/BJ20051451. PubMed PMID: 16396630; PubMed Central PMCID: PMCPMC1422764.
34. Marcus SL, Knodler LA, Finlay BB. Salmonella enterica serovar Typhimurium effector SigD/SopB is membrane-associated and ubiquitinated inside host cells. Cell Microbiol. 2002;4(7):435-46. PubMed PMID: 12102689.
35. Cain RJ, Hayward RD, Koronakis V. The target cell plasma membrane is a critical interface for Salmonella cell entry effector-host interplay. Mol Microbiol. 2004;54(4):887-904. doi: 10.1111/j.1365-2958.2004.04336.x. PubMed PMID: 15522075.
36. Burkinshaw BJ, Prehna G, Worrall LJ, Strynadka NC. Structure of Salmonella effector protein SopB N-terminal domain in complex with host Rho GTPase Cdc42. J Biol Chem. 2012;287(16):13348-55. Epub 2012/02/23. doi: 10.1074/jbc.M111.331330. PubMed PMID: 22362774; PubMed Central PMCID: PMCPMC3339929.
37. Vonaesch P, Sellin ME, Cardini S, Singh V, Barthel M, Hardt WD. The Salmonella Typhimurium effector protein SopE transiently localizes to the early SCV and contributes to intracellular replication. Cell Microbiol. 2014;16(12):1723-35. Epub 2014/08/26. doi: 10.1111/cmi.12333. PubMed PMID: 25052734.

38. Stender S, Friebel A, Linder S, Rohde M, Mirold S, Hardt WD. Identification of SopE2 from Salmonella typhimurium, a conserved guanine nucleotide exchange factor for Cdc42 of the host cell. Mol Microbiol. 2000;36(6):1206-21. PubMed PMID: 10931274.
39. Salcedo SP, Holden DW. SseG, a virulence protein that targets Salmonella to the Golgi network. EMBO J. 2003;22(19):5003-14. doi: 10.1093/emboj/cdg517. PubMed PMID: 14517239; PubMed Central PMCID: PMCPMC204495.
40. Yu XJ, Liu M, Holden DW. Salmonella Effectors SseF and SseG Interact with Mammalian Protein ACBD3 (GCP60) To Anchor Salmonella-Containing Vacuoles at the Golgi Network. MBio. 2016;7(4). Epub 2016/07/12. doi: 10.1128/mBio.00474-16. PubMed PMID: 27406559; PubMed Central PMCID: PMCPMC4958240.
41. Ruiz-Albert J, Yu XJ, Beuzón CR, Blakey AN, Galyov EE, Holden DW. Complementary activities of SseJ and SifA regulate dynamics of the Salmonella typhimurium vacuolar membrane. Mol Microbiol. 2002;44(3):645-61. PubMed PMID: 11994148.
42. LaRock DL, Brzovic PS, Levin I, Blanc MP, Miller SI. A Salmonella typhimurium-translocated glycerophospholipid:cholesterol acyltransferase promotes virulence by binding to the RhoA protein switch regions. J Biol Chem. 2012;287(35):29654-63. Epub 2012/06/27. doi: 10.1074/jbc.M112.363598. PubMed PMID: 22740689; PubMed Central PMCID: PMCPMC3436183.
43. Freeman JA, Ohl ME, Miller SI. The Salmonella enterica serovar typhimurium translocated effectors SseJ and SifB are targeted to the Salmonella-containing vacuole. Infect Immun. 2003;71(1):418-27. PubMed PMID: 12496192; PubMed Central PMCID: PMCPMC143161.
44. Domingues L, Ismail A, Charro N, Rodríguez-Escudero I, Holden DW, Molina M, et al. The Salmonella effector SteA binds phosphatidylinositol 4-phosphate for subcellular targeting within host cells. Cell Microbiol. 2016;18(7):949-69. Epub 2016/03/11. doi: 10.1111/cmi.12558. PubMed PMID: 26676327.
45. Nougayrède JP, Donnenberg MS. Enteropathogenic Escherichia coli EspF is targeted to mitochondria and is required to initiate the mitochondrial death pathway. Cell Microbiol. 2004;6(11):1097-111. doi: 10.1111/j.1462-5822.2004.00421.x. PubMed PMID: 15469437.
46. Alto NM, Weflen AW, Rardin MJ, Yarar D, Lazar CS, Tonikian R, et al. The type III effector EspF coordinates membrane trafficking by the spatiotemporal activation of two eukaryotic signaling pathways. J Cell Biol. 2007;178(7):1265-78. doi: 10.1083/jcb.200705021. PubMed PMID: 17893247; PubMed Central PMCID: PMCPMC2064658.
47. Nougayrède JP, Foster GH, Donnenberg MS. Enteropathogenic Escherichia coli effector EspF interacts with host protein Abcf2. Cell Microbiol. 2007;9(3):680-93. Epub 2006/10/25. doi: 10.1111/j.1462-5822.2006.00820.x. PubMed PMID: 17064289.
48. Marchès O, Batchelor M, Shaw RK, Patel A, Cummings N, Nagai T, et al. EspF of enteropathogenic Escherichia coli binds sorting nexin 9. J Bacteriol. 2006;188(8):3110-5. doi: 10.1128/JB.188.8.3110-3115.2006. PubMed PMID: 16585770; PubMed Central PMCID: PMCPMC1447016.
49. Campellone KG, Cheng HC, Robbins D, Siripala AD, McGhie EJ, Hayward RD, et al. Repetitive N-WASP-binding elements of the enterohemorrhagic Escherichia coli effector EspF(U) synergistically activate actin assembly. PLoS Pathog. 2008;4(10):e1000191. Epub 2008/10/31. doi: 10.1371/journal.ppat.1000191. PubMed PMID: 18974829; PubMed Central PMCID: PMCPMC2567903.
50. Weiss SM, Ladwein M, Schmidt D, Ehinger J, Lommel S, Städing K, et al. IRSp53 links the enterohemorrhagic E. coli effectors Tir and EspFU for actin pedestal formation. Cell Host Microbe. 2009;5(3):244-58. doi: 10.1016/j.chom.2009.02.003. PubMed PMID: 19286134.

51. Vingadassalom D, Kazlauskas A, Skehan B, Cheng HC, Magoun L, Robbins D, et al. Insulin receptor tyrosine kinase substrate links the E. coli O157:H7 actin assembly effectors Tir and EspF(U) during pedestal formation. Proc Natl Acad Sci U S A. 2009;106(16):6754-9. Epub 2009/04/06. doi: 10.1073/pnas.0809131106. PubMed PMID: 19366662; PubMed Central PMCID: PMCPMC2672544.
52. Tu X, Nisan I, Yona C, Hanski E, Rosenshine I. EspH, a new cytoskeleton-modulating effector of enterohaemorrhagic and enteropathogenic Escherichia coli. Mol Microbiol. 2003;47(3):595-606. PubMed PMID: 12535063.
53. Dong N, Liu L, Shao F. A bacterial effector targets host DH-PH domain RhoGEFs and antagonizes macrophage phagocytosis. EMBO J. 2010;29(8):1363-76. Epub 2010/03/18. doi: 10.1038/emboj.2010.33. PubMed PMID: 20300064; PubMed Central PMCID: PMCPMC2868573.
54. Kurushima J, Nagai T, Nagamatsu K, Abe A. EspJ effector in enterohemorrhagic E. coli translocates into host mitochondria via an atypical mitochondrial targeting signal. Microbiol Immunol. 2010;54(7):371-9. doi: 10.1111/j.1348-0421.2010.00218.x. PubMed PMID: 20618683.
55. Young JC, Clements A, Lang AE, Garnett JA, Munera D, Arbeloa A, et al. The Escherichia coli effector EspJ blocks Src kinase activity via amidation and ADP ribosylation. Nat Commun. 2014;5:5887. Epub 2014/12/19. doi: 10.1038/ncomms6887. PubMed PMID: 25523213; PubMed Central PMCID: PMCPMC4284639.
56. Hemrajani C, Berger CN, Robinson KS, Marchès O, Mousnier A, Frankel G. NleH effectors interact with Bax inhibitor-1 to block apoptosis during enteropathogenic Escherichia coli infection. Proc Natl Acad Sci U S A. 2010;107(7):3129-34. Epub 2010/01/26. doi: 10.1073/pnas.0911609106. PubMed PMID: 20133763; PubMed Central PMCID: PMCPMC2840288.
57. Gao X, Wan F, Mateo K, Callegari E, Wang D, Deng W, et al. Bacterial effector binding to ribosomal protein s3 subverts NF-kappaB function. PLoS Pathog. 2009;5(12):e1000708. Epub 2009/12/24. doi: 10.1371/journal.ppat.1000708. PubMed PMID: 20041225; PubMed Central PMCID: PMCPMC2791202.
58. Pham TH, Gao X, Singh G, Hardwidge PR. Escherichia coli virulence protein NleH1 interaction with the v-Crk sarcoma virus CT10 oncogene-like protein (CRKL) governs NleH1 inhibition of the ribosomal protein S3 (RPS3)/nuclear factor κB (NF-κB) pathway. J Biol Chem. 2013;288(48):34567-74. Epub 2013/10/21. doi: 10.1074/jbc.M113.512376. PubMed PMID: 24145029; PubMed Central PMCID: PMCPMC3843070.
59. Kenny B, Finlay BB. Intimin-dependent binding of enteropathogenic Escherichia coli to host cells triggers novel signaling events, including tyrosine phosphorylation of phospholipase C-gamma1. Infect Immun. 1997;65(7):2528-36. PubMed PMID: 9199415; PubMed Central PMCID: PMCPMC175357.
60. Luo Y, Frey EA, Pfuetzner RA, Creagh AL, Knoechel DG, Haynes CA, et al. Crystal structure of enteropathogenic Escherichia coli intimin-receptor complex. Nature. 2000;405(6790):1073-7. doi: 10.1038/35016618. PubMed PMID: 10890451.
61. Haenssler E, Ramabhadran V, Murphy CS, Heidtman MI, Isberg RR. Endoplasmic Reticulum Tubule Protein Reticulon 4 Associates with the Legionella pneumophila Vacuole and with Translocated Substrate Ceg9. Infect Immun. 2015;83(9):3479-89. Epub 2015/06/22. doi: 10.1128/IAI.00507-15. PubMed PMID: 26099580; PubMed Central PMCID: PMCPMC4534651.
62. Aurass P, Schlegel M, Metwally O, Harding CR, Schroeder GN, Frankel G, et al. The Legionella pneumophila Dot/Icm-secreted effector PlcC/CegC1 together with PlcA and PlcB promotes virulence and belongs to a novel zinc metallophospholipase C family present in bacteria and fungi. J Biol Chem. 2013;288(16):11080-92. Epub 2013/03/01. doi: 10.1074/jbc.M112.426049. PubMed PMID: 23457299; PubMed Central PMCID: PMCPMC3630882.

63. de Felipe KS, Glover RT, Charpentier X, Anderson OR, Reyes M, Pericone CD, et al. Legionella eukaryotic-like type IV substrates interfere with organelle trafficking. PLoS Pathog. 2008;4(8):e1000117. Epub 2008/08/01. doi: 10.1371/journal.ppat.1000117. PubMed PMID: 18670632; PubMed Central PMCID: PMCPMC2475511.
64. Shi X, Halder P, Yavuz H, Jahn R, Shuman HA. Direct targeting of membrane fusion by SNARE mimicry: Convergent evolution of Legionella effectors. Proc Natl Acad Sci U S A. 2016;113(31):8807-12. Epub 2016/07/19. doi: 10.1073/pnas.1608755113. PubMed PMID: 27436892; PubMed Central PMCID: PMCPMC4978295.
65. Mousnier A, Schroeder GN, Stoneham CA, So EC, Garnett JA, Yu L, et al. A new method to determine in vivo interactomes reveals binding of the Legionella pneumophila effector PieE to multiple rab GTPases. MBio. 2014;5(4). Epub 2014/08/12. doi: 10.1128/mBio.01148-14. PubMed PMID: 25118235; PubMed Central PMCID: PMCPMC4145681.
66. Hsu F, Zhu W, Brennan L, Tao L, Luo ZQ, Mao Y. Structural basis for substrate recognition by a unique Legionella phosphoinositide phosphatase. Proc Natl Acad Sci U S A. 2012;109(34):13567-72. Epub 2012/08/07. doi: 10.1073/pnas.1207903109. PubMed PMID: 22872863; PubMed Central PMCID: PMCPMC3427105.
67. Banga S, Gao P, Shen X, Fiscus V, Zong WX, Chen L, et al. Legionella pneumophila inhibits macrophage apoptosis by targeting pro-death members of the Bcl2 protein family. Proc Natl Acad Sci U S A. 2007;104(12):5121-6. Epub 2007/03/14. doi: 10.1073/pnas.0611030104. PubMed PMID: 17360363; PubMed Central PMCID: PMCPMC1829273.
68. Franco IS, Shohdy N, Shuman HA. The Legionella pneumophila effector VipA is an actin nucleator that alters host cell organelle trafficking. PLoS Pathog. 2012;8(2):e1002546. Epub 2012/02/23. doi: 10.1371/journal.ppat.1002546. PubMed PMID: 22383880; PubMed Central PMCID: PMCPMC3285593.
69. Degtyar E, Zusman T, Ehrlich M, Segal G. A Legionella effector acquired from protozoa is involved in sphingolipids metabolism and is targeted to the host cell mitochondria. Cell Microbiol. 2009;11(8):1219-35. Epub 2009/04/27. doi: 10.1111/j.1462-5822.2009.01328.x. PubMed PMID: 19438520.
70. Okujava R, Guye P, Lu YY, Mistl C, Polus F, Vayssier-Taussat M, et al. A translocated effector required for Bartonella dissemination from derma to blood safeguards migratory host cells from damage by co-translocated effectors. PLoS Pathog. 2014;10(6):e1004187. Epub 2014/06/19. doi: 10.1371/journal.ppat.1004187. PubMed PMID: 24945914; PubMed Central PMCID: PMCPMC4063953.
71. Ohya K, Handa Y, Ogawa M, Suzuki M, Sasakawa C. IpgB1 is a novel Shigella effector protein involved in bacterial invasion of host cells. Its activity to promote membrane ruffling via Rac1 and Cdc42 activation. J Biol Chem. 2005;280(25):24022-34. Epub 2005/04/22. doi: 10.1074/jbc.M502509200. PubMed PMID: 15849186.
72. Handa Y, Suzuki M, Ohya K, Iwai H, Ishijima N, Koleske AJ, et al. Shigella IpgB1 promotes bacterial entry through the ELMO-Dock180 machinery. Nat Cell Biol. 2007;9(1):121-8. Epub 2006/12/17. doi: 10.1038/ncb1526. PubMed PMID: 17173036.
73. Niebuhr K, Giuriato S, Pedron T, Philpott DJ, Gaits F, Sable J, et al. Conversion of PtdIns(4,5)P(2) into PtdIns(5)P by the S.flexneri effector IpgD reorganizes host cell morphology. EMBO J. 2002;21(19):5069-78. PubMed PMID: 12356723; PubMed Central PMCID: PMCPMC129044.
